# Evidence for Phosphorylation-Dependent, Dynamic, Regulation of mGlu5 and Homer2 in Expression of Cocaine Aversion in Mice

**DOI:** 10.1523/ENEURO.0423-22.2023

**Published:** 2023-04-20

**Authors:** Karen K. Szumlinski, Jacqueline Beltran, Eliyana van Doren, C. Leonardo Jimenez Chavez, Racquel D. Domingo-Gonzalez, Cindy M. Reyes, Alexis W. Ary, Andrew Lang, Weiruo Guo, Paul F. Worley, Kimberly M. Huber

**Affiliations:** 1 Department of Psychological and Brain Sciences, University of California Santa Barbara, Santa Barbara, CA 93106; 2 Department of Molecular, Cellular and Developmental Biology and the Neuroscience Research Institute, University of California Santa Barbara, Santa Barbara, CA 93106; 3 Department of Neuroscience, O’Donnell Brain Institute, University of Texas Southwestern Medical Center, Dallas, TX 75390; 4 Department of Neuroscience, Johns Hopkins University School of Medicine, Baltimore, MD 21205

**Keywords:** anxiety, cocaine, emotionality, Homer proteins, mGlu5

## Abstract

Cocaine-induced changes in the expression of the glutamate-related scaffolding protein Homer2 influence this drug’s psychostimulant and rewarding properties. In response to neuronal activity, Homer2 is phosphorylated on S117/S216 by calcium-calmodulin kinase IIα (CaMKIIα), which induces a rapid dissociation of mGlu5-Homer2 scaffolds. Herein, we examined the requirement for Homer2 phosphorylation in cocaine-induced changes in mGlu5-Homer2 coupling, to include behavioral sensitivity to cocaine. For this, mice with alanine point mutations at (S117/216)-Homer2 (*Homer2^AA/AA^*) were generated, and we determined their affective, cognitive and sensorimotor phenotypes, as well as cocaine-induced changes in conditioned reward and motor hyperactivity. The *Homer2^AA/AA^* mutation prevented activity-dependent phosphorylation of S216 Homer2 in cortical neurons, but *Homer2^AA/AA^* mice did not differ from wild-type (WT) controls with respect to Morris maze performance, acoustic startle, spontaneous or cocaine-induced locomotion. *Homer2^AA/AA^* mice exhibited signs of hypoanxiety similar to the phenotype of transgenic mice with a deficit in signal-regulated mGluR5 phosphorylation (*Grm5^AA/AA^*). However, opposite of *Grm5^AA/AA^* mice, *Homer2^AA/AA^* mice were less sensitive to the aversive properties of high-dose cocaine under both place-conditioning and taste-conditioning procedures. Acute injection with cocaine caused dissociation of mGluR5 and Homer2 in striatal lysates from WT, but not *Homer2^AA/AA^* mice, suggesting a molecular basis for the deficit in cocaine aversion. These findings indicate that CaMKIIα-dependent phosphorylation of Homer2 gates the negative motivational valence of high-dose cocaine via regulation of mGlu5 binding, furthering an important role for dynamic changes in mGlu5-Homer interactions in addiction vulnerability.

## Significance Statement

Globally, psychostimulant use has again risen to reach epidemic proportions, particularly in the United States. However, we continue to face a knowledge gap regarding the biological bases of psychostimulant addiction vulnerability to inform disease prognosis and treatment-based recovery. Herein, we show that the psychomotor stimulant cocaine induces the uncoupling of the mGlu5 glutamate receptor from its scaffolding protein Homer2 in brain. Using a transgenic mouse model with deficits cocaine-induced uncoupling of mGlu5-Homer2, we demonstrate an important role for Homer2 scaffolding of mGlu5 in regulating cocaine’s aversive properties, without influencing cocaine reward. Findings suggest that environmental factors, to include cocaine exposure, that affect mGlu5-Homer2 scaffolding dynamics may contribute to an individual’s subjective response to cocaine to influence addiction vulnerability.

## Introduction

Cocaine remains one of the most widely used illicit drugs worldwide, with ∼18.2 million people reporting cocaine use (United Nations Office on Drugs and Crime, 2021). Despite this, the knowledge gap regarding the neurobiological substrates underlying cocaine use disorder impedes therapeutic progress. Cocaine impacts glutamate transmission in brain, producing region-selective changes in the expression and function of the Group1 metabotropic glutamate receptor subtype mGlu5 (cf. Niedzielska-Andres et al., 2021), which contribute to cocaine-seeking behavior in animal models (Gass and Olive, 2008; Ben-Shahar et al., 2013; Knackstedt and Schwendt, 2016; Gobin, et al., 2020). mGlu5 receptors act primarily via Gq/11 proteins to initiate the PLC/IP3/Ca^2+^ cascade (Niswender and Conn, 2010; Iacovelli et al., 2013), as well as signaling to mitogen-activated protein kinase pathways (Wang et al., 2007), and PI3K/Akt/mTOR signaling (Rong et al., 2003; Ronesi and Huber, 2008).

mGlu5 localization and signaling is regulated by Homer scaffolding proteins (Tu et al., 1998). In mammals, Homer proteins are encoded by three genes (*Homer1*, *Homer2*, *Homer3*; Soloviev et al., 2000), which bind mGlu5 and tetramerize through their C-terminus coiled-coil domains to form signaling scaffolds with mGlu5 and its effectors such as the IP3 receptor, PI3K Enhancer and NMDA receptors. Mutations in mGlu5 that prevent Homer binding (F1128 to R; *Grm5^R/R^*) result in reduced mGlu5 in the postsynaptic density and constitutive signaling, as well as loss of agonist-induced signaling to downstream effectors such as ERK and mTOR. Both a truncated, dominant negative, isoform Homer1a and long Homers are regulated in brain by cocaine or its absence (cf. Szumlinski et al., 2008; Marton et al., 2015). Further, *Homer1* and *Homer2* gene products regulate cocaine’s psychomotor-activating, rewarding and reinforcing properties in rodent models (Swanson et al., 2001; Szumlinski et al., 2004; Knackstedt et al., 2010; Ary et al., 2013; Gould et al., 2015; Datko et al., 2017).

mGlu5 binding with Homers and other interactors are dynamic and highly regulated. Acute cocaine induces ERK phosphorylation of (T1123/S1126)-mGlu5, which promotes binding of the prolyl isomerase Pin1, and the isomerization of the C-terminus of mGlu5 to enhance mGlu5 signaling to NMDARs (Park et al., 2013). Mice with site-specific mutations that prevent phosphorylation of (T1123/S1126)-mGlu5 (*GRM5^AA/AA^* mice) have deficits in dopamine-induced regulation of mGlu5 signaling to NMDARs, fail to develop cocaine-induced sensitization (Park et al., 2013), but exhibit a very robust cocaine-conditioned place-aversion (Campbell et al., 2019). These latter findings implicate phosphorylation-dependent, dynamical interactions between mGlu5 and constitutively expressed Homer proteins in regulating both the psychomotor-activating and rewarding properties of cocaine.

Brief depolarization of neurons and Ca^2+^ influx through both NMDARs and Ca^2+^ channels induce phosphorylation of Homers by Ca^2+^/calmodulin-dependent protein kinase IIα (CaMKIIα). All three Homers have CaMKIIα phosphorylation sites within their “hinge” region between the EVH1 and coiled-coil domains and phosphorylation of these sites results in reduced binding to mGlu5, as well as other Homer interactors (Mizutani et al., 2008; Guo et al., 2015). Both Homer1 and Homer2 share a conserved CaMKIIα phosphorylation site at S117, with Homer2 possessing an additional phosphorylation site at S216. In the case of Homer2, CaMKIIα-dependent phosphorylation of (S117/S216)-Homer2 rapidly dissociates mGlu5-Homer scaffolds (Guo et al., 2015). To date, the biopsychological implications of CaMKIIα-dependent Homer phosphorylation have been inferred from studies of the *Fmr1* knock-out (KO) mouse model of Fragile X syndrome, which exhibits elevated CaMKIIα activity, Homer1 and Homer2 hyperphosphorylation and reduced Homer-mGlu5 binding (Guo et al., 2015), which can be rescued by reducing CaMKIIα activity or levels or replacement with mutant Homers that cannot be phosphorylated by CaMKIIα (Guo et al., 2015). Further, *Fmr1* KO mice exhibit blunted cocaine-induced motor sensitization and conditioned reward, which can be rescued by lowering mGlu5 expression (Smith et al., 2014). These results reveal an alternate, Homer-directed, phosphorylation-dependent mechanism for controlling Homer-mGlu5 scaffolding, neuronal excitability and perhaps also cocaine-induced behavioral plasticity. Indeed, CaMKIIα hyperactivity has long been implicated in cocaine addiction-related behavior and neuroplasticity (cf. García-Pardo et al., 2016; Yu et al., 2017). Here, we show that acute cocaine activates CaMKIIα in mouse brain and dissociates Homer2-mGlu5 scaffolds, the latter of which is absent in a knock-in (KI) mouse with alanine substitutions at (S117/S216)-Homer2 that prevent its phosphorylation by CaMKIIα (*Homer2^AA/AA^* mouse). *Homer2^AA/AA^* mice exhibit certain behavioral signs of hypoanxiety that are similar to those expressed by *GRM5^R/R^* KI mice with reduced steady state Homer binding and *GRM5^AA/AA^* KI mice with deficient regulated mGlu5 interactions. However, opposite *GRM5^AA/AA^* mice (Campbell et al., 2019), *Homer2^AA/AA^* mice are resilient to the conditioned aversive properties of cocaine. This work provides knowledge of the dynamic regulation of mGluR5-Homer2 scaffolds by cocaine that actively gates the negative affective valence of this highly addictive substance.

## Materials and Methods

### Subjects

#### *Homer2^AS117A/S120A^* KI mouse

To determine the functional consequences of CaMKIIα-dependent phosphorylation of Homer2 *in vivo*, a KI mouse with serine (S) →alanine (A) point mutations at amino acid positions 117 and 120 of Homer2 was generated using CRISPR/Cas9 and microinjection single-cell C57BL/6J embryos at the Transgenic Technology Center at Institution 1. Two gRNAs (S117A site: CTAGCCAGAGACAAGTCCCAGG; S216 site: CAGCGTGGAGCAGTGGAAGCGG) were designed and validated by Sigma-Aldrich. The sequence of the oligo DNA donor for S117A knock-in is: 5′-aagtaagtcctaataactgttccatatgttcagtttgcagagaagttccaggaggtaagagaagctgccaggctagccagagacaaggcccaagagaaaaccgagacctccagcaatcattcccaagtaagtaagtcatttgtccccgagatgcatctgagtgtgatcagtaattgagat-3′. The sequence of the oligo DNA donor for S216A knock-in is: 5′-gatggagctgcagaccctgcgggagagcaacgcccggctgaccacggcactgcaggagtcggcggccagcgtggagcagtggaagcgacagttcgccatctgcagggacgagaatgacaggctccgcagcaaggtgggcctcggcagccagcgggacagagggcctggccgactggagca-3′.

**Figure 1.**
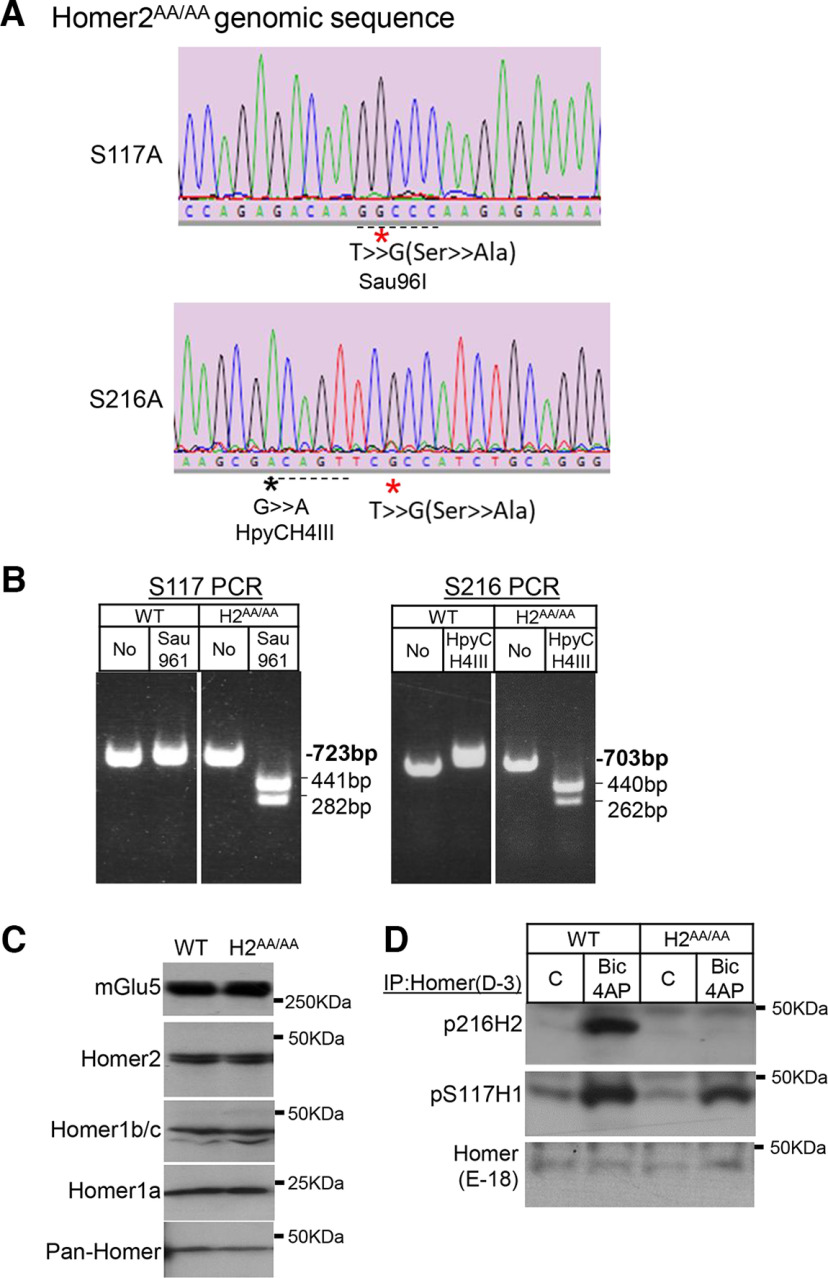
Generation of Homer2 S117A, S216A (*Homer2^AA/AA^*) mutant knock-in mouse. ***A***, Sequencing of Homer2 S117A site and S216A site PCR-amplified genomic DNA from homozygote *Homer2^AA/AA^* mutant mouse. Asterisks indicate mutants. The dotted underlines indicate knock-in-created Sau96I(S117A) and HpyCH4III(S216A) restriction enzyme sites for genotyping. ***B***, PCR genotyping of wild-type (WT) and homozygous *Homer2^AA/AA^* mutant mouse. The PCR product images of WT and homozygous *Homer2^AA/AA^* mutant after Sau96I and HpyCH4III digestion. ***C***, Western blots of cortical homogenates from WT and *Homer2^AA/AA^* mutant mice demonstrate normal levels of mGlu5 and Homers 1 and 2. ***D***, Brief, 5-min, treatment of mouse neocortical slices with bicuculline and 4-aminopyridine (4-AP) induced phosphorylation (P) of S216 Homer2 and S117 Homer1 in WT mice as detected with phosphorylation site antibodies after immunoprecipitation with a pan-Homer antibody. In neocortical slices from *Homer2^AA/AA^* mice, P-S216 Homer2 was not detected, but normal activity-induced p(S117)-Homer1 was observed.

Genotyping of *Homer2^S117A/S120A^* KI (*Homer2^AA/AA^*) mice was performed with the specific primers (H2S117AF: 5′-AGT TGC AGG TGT GCA CAT GGC A-3′ and H2S117AR: 5′-AGG TGA CTC CGA TTG CAT CAG T-3′; H2S216AF: 5′-ACCCAACATGAGAGAGCTGAC-3′′ and H2S216AR: 5′-CAGGGTCTCTGTGGAAACATACTC-3′′). PCR products were digested by the restriction enzyme Sau96I for H2S117A and HpyCH4III for H2S216A, the PCR products from the mutant allele was sensitive to Sau96I and HpyCH4III (Fig. 1). *Homer2^AA/AA^* were backcrossed to C57BL/6J mice for two to five generations before experiments.

For all experiments, both female and male wild-type (WT) and homozygous *Homer2^AA/AA^* littermate mice were generated at Institution 1 from heterozygous breeder pairs. For behavioral experiments, between five and eight weeks of age, WT and *Homer2^AA/AA^* littermates were relocated to Institution 2, where they were quarantined under standard housing conditions on a ventilated rack for a maximum of nine weeks. Upon health clearance, mice were relocated to a standard holding room in the main vivarium space. Mice were initially housed under a regular 12/12 h light/dark cycle (lights on: 7 A.M.), with testing conducted between 8 A.M. and 5 P.M. The subset of mice slated for taste-conditioning studies were then re-located to a distinct holding room under a 12/12 h reverse light/dark cycle (lights on: 10 P.M.) and were allowed to acclimate for 10 d, before testing. In all, two distinct cohorts of WT and *Homer2^AA/AA^* mice were employed in these studies, spaced approximately six months apart. Each cohort consisted of approximately equal numbers of male and female wild-type (WT) and *Homer2^AA/AA^* mice (± three mice).

#### *GRM5^F1128R^* KI mouse

For comparison, the effects of preventing Homer-mGlu5 scaffolding on spatial learning/memory and cocaine-conditioned reward were also determined using the *GRM5^F1128R^* transgenic mouse (*GRM5^R/R^*). This mouse houses a single F→R point mutation at F1128 that lies within the Homer binding domain on mGlu5 that prevents Homer binding to the receptor (Cozzoli et al., 2009). Thus, the consequences of the *GRM5^R/R^* mutation are functionally opposite to that of the *Homer2^AA/AA^* mutation. This particular mGlu5 mutant exhibits a modest potentiation of cocaine-induced behavioral sensitization (Park et al., 2013), reduced binge alcohol-drinking (Cozzoli et al., 2009, 2012), in addition to affective and sensorimotor phenotypes similar to those reported in *Fmr1* KO mice (Guo et al., 2016). The effects of disrupting Homer-mGlu5 binding on cocaine reward and learning/memory have not been reported. Thus, female and male WT and *GRM5^R/R^* mice were generated by the mating of heterozygous breeder pairs. Behavioral testing was conducted both female and male adult (six to eight weeks of age) littermate offspring, with approximately equal numbers of males and females employed for each genotype (± three mice).

#### *GRM5^T1123A/S1126A^* KI mouse

Activity-dependent mGlu5 phosphorylation by proline-directed kinases within the Homer binding domain increases the binding avidity of Homer-mGlu5 scaffolds (Orlando et al., 2009; Hu et al., 2012; Park et al., 2013). *GRM5^T1123A/S1126A^* transgenic mice (*GRM5^AA/AA^*), in which this phosphorylation is prevented, exhibit reduced mGlu5-Homer binding avidity, concomitant with blunted cocaine-induced locomotor and neurochemical sensitization (Park et al., 2013), as well as robust cocaine-conditioned place-aversion and a hypoanxious, “*Fmr1* KO-like,” affective phenotype (Campbell et al., 2019). As the consequences of the *GRM5^AA/AA^* mutation for Homer-mGlu5 scaffolding are also predicted to be functionally opposite that produced by the *Homer2^AA/AA^* mutation, we compared, in certain assays, female and male littermate WT and *GRM5^AA/AA^* mice, derived from a colony of heterozygous breeders. As for *GRM5^R/R^* mice, behavioral testing was conducted in adult mice (six to eight weeks of age), with approximately equal number of males and females employed for each genotype (± three to four mice).

Experimental mice were housed in same-sex groups of two to four in an Association for Assessment and Accreditation of Laboratory Animal Care-approved animal facility in standard mouse cages housed on ventilated racks under a regular 12/12 h light/dark cycle (lights on: 7 A.M.). All testing was conducted during the light cycle with food and water were available *ad libitum*, unless otherwise indicated. All experiments were approved by the Institutional Animal Care and Use Committees of our respective institutions and were conducted in accordance with the National Institutes of Health Principles of Laboratory Animal Care.

### Neocortical slice preparation and treatment

Neocortical slices were prepared from postnatal day (P)28–P30 mice. Briefly, mice were anesthetized with ketamine (125 mg/kg)/xylazine (25 mg/kg) and transcardially perfused with chilled (4°C) sucrose dissection buffer containing the following (in mm): 2.6 KCl, 1.25 NaH_2_PO_4_, 26 NaHCO_3_, 0.5 CaCl_2_, 5 MgCl_2_, 212 sucrose, and 10 dextrose aerated with 95% O_2_/5% CO_2_. Coronal neocortical slices were obtained on a Leica VT1000S slicer. Slices recovered for 3.5 h and were maintained at 32°C in artificial cerebrospinal fluid containing (in mm): 119 NaCl, 2.5 KCl, 2 CaCl_2_, 1 MgCl_2_, 26 NaHCO_3_, 1 NaH_2_PO_4_, and 11 D-glucose aerated with 95% O_2_/5% CO_2_ to pH 7.4. Slices were then treated with bicuculline (100 μm) and 4-aminopyridine (4-AP; 100 μm) for 5 min.

### Co-immunoprecipitation and Western blotting

To examine for cocaine-induced changes in Homer2 phosphorylation and mGlu5 binding, adult mice were injected intraperitoneally with saline or cocaine dissolved in saline at 2 mg/ml and 20 mg/kg dosing. Mice were then returned to the animal colony room in their home cage for 1 h. Mice were then rapidly decapitated and whole striatum was dissected and immediately frozen in liquid nitrogen.

Tissue was lysed with co-immunoprecipitation (co-IP) buffer (50 mm Tris, pH 7.4, 120 mm NaCl, 50 mm NaF, and 1% Triton X-100), containing protease inhibitor cocktail (Sigma), phosphatase inhibitor cocktail 2 and 3 (Sigma). The striatum samples were sonicated and centrifuged (10 min at 13,000 × *g*) to remove unlysed tissue. Protein levels in the supernatant were measured using Pierce BCA kit and 200 μg protein were incubated with 1 μg of pan-Homer antibody (Santa Cruz Biotechnology, D-3; sc-17842; RRID:AB_627742) overnight at 4°C and then with protein A/G bead slurry (Thermo Scientific) was added for one additional hour. Beads were then washed with co-immunoprecipitation buffer three times. Samples were subjected to 12% SDS-PAGE gel analysis followed by Western blotting. Antibodies used in this study for Western blotting include: pan-Homer (Santa Cruz Biotechnology, E-18; sc-8921; RRID:AB_648368), mGlu5 (Millipore; 6451; no RRID available), Homer2 (Synaptic Systems; 160203; RRID:AB_10807099), GAPDH (Millipore; MAB374; RRID:AB_2107445), pT286CaMKIIα (Sigma; SAB4504356; no RRID available), anti-CaMKIIα (Santa Cruz; sc-5391; RRID:AB_634553), Homer1 phospho-S117 Homer2 phospho-S216 (Guo et al., 2015).

### General experimental design of the behavioral studies

To determine the behavioral phenotype of *Homer2^AA/AA^* mice, their behavior was compared with that of WT animals and all experiments included the between-subjects factor of Genotype. The majority of behavioral experiments included approximately equal numbers of male and female mice and also included the between-subjects factor of Sex. The majority of behavioral outcomes involved a single dependent variable and, thus, were analyzed using univariate ANOVAs. For experiments involving repeated measures, the statistical analyses also included a within-subjects factor. When a sex difference was not detected in the initial statistical analyses, the data were collapsed across the Sex factor for re-analysis of Genotype effects and interactions, when applicable. For comparison, some behavioral experiments were also conducted in *GRM5^R/R^* and *GRM5^AA/AA^* mutant mice and compared with their distinct WT controls. The general experimental design and statistical approaches employed in the study of mGlu5-related mutant mice were similar to those employed for *Homer2^AA/AA^* versus their WT mice. The specific statistical approach to each dataset is detailed for each paradigm in the subsections below.

### Prepulse inhibition of acoustic startle

*GRM5^R/R^* mice with disrupted Homer-mGlu5 binding exhibit impaired prepluse inhibition (PPI) of acoustic startle, in a manner consistent with *Fmr1* KO mice that exhibit Homer2 hyperphosphorylation and dissociated Homer-mGlu5 scaffolds (Guo et al., 2016). To determine whether increasing the avidity of Homer-mGlu5 binding by preventing phosphorylation-dependent uncoupling of Homer2-mGlu5 scaffolds also impacts sensorimotor processing, we compared female and male WT and *Homer2^AA/AA^* mice in a standard PPI paradigm. For comparison, we compared acoustic startle and PPI also between *GRM5^AA/AA^* phospho-mutant mice with reduced mGlu5-Homer binding avidity.

Six trial types were conducted: startle pulse (st110, 110 dB/40 ms), low prepulse stimulus alone (st74, 74 dB/20 ms), high prepulse stimulus alone (st90, 90 dB/20 ms), low or high prepulse stimulus played 100 ms before the onset of the startle pulse (pp74 and pp90, respectively), and no acoustic stimulus (st0; only background noise). All trials were presented in a randomized order; st0, st110, pp74, and pp90 trials were administered 10 times each, whereas st74 and st90 were presented five times each. Background noise in each chamber was 70 dB, and the average intertrial interval lasted 15 s. The data for each startle stimulus were analyzed using a Genotype × Sex × Stimulus ANOVA, with repeated measures on the Stimulus factor (four levels). The % PPI by the 74 and 90 dB prepulses was calculated as the percent change in the average startle during st110 trials during the pp74 and pp90 trials and analyzed using a Genotype × Sex × Prepulse ANOVA, with repeated measures on the Prepulse factor (two levels).

### Morris water maze

*Frm1* KO mice with dissociated Homer-mGlu5 scaffolds exhibit learning and memory impairments in a variety of assays, including those of spatial learning and memory (cf. Santos et al., 2014). To examine how different Homer-mGlu5 scaffolding mutations impact cognitive function, genotypic differences in spatial reference learning and memory were examined in a Morris water maze paradigm. A stainless steel circular tank (200 cm in diameter, 60 cm in height) served as the maze, with salient intramaze cues located at all four compass points on the inside walls of the maze and extra-maze cues present in the testing room. The tank was filled with room-temperature water to a depth of 50 cm. To ensure similar swim performance and visual ability, Morris maze training began with a test in which a visible flag was attached to the clear platform (protruding 20 cm out of the water) and the platform was placed in the NW quadrant. The mice were released from the opposite quadrant (i.e., SE) and allowed 2 min to locate the flagged platform. All animals successfully located the flagged platform and so only one flag test was performed for each study.

Over the course of the next 4 d, the flag was removed from the platform so the platform was no longer visible and the invisible platform remained in the NE quadrant (i.e., a quadrant distinct from the Flag Test). Each day, mice underwent 4 trials, in which they were randomly placed in the pool at each of the four compass points and swimming was recorded digitally by a video camera mounted on the ceiling directly above the tank for a period of 2 min/trail (ANY-Maze, Stoelting). If the mice were unable to locate the platform during the allotted time, they were guided to the platform where they remained for 30 s. The mice were trained in series for each of the four release points; as such, the intertrial interval was ∼30–45 min. The release points were randomized across the 4 d of training (total of 16 training trials). At 24 h following the last training day, a memory probe test was performed, in which the platform was removed from the tank and swimming location recorded over a 2-min period. In the study of *Homer2^AA/AA^* mice, the day following the probe test, reversal learning was assayed by placing the hidden platform in the SW quadrant (i.e., the quadrant opposite that during the training phase) and mice underwent 4 trials (one per compass location) to locate the relocated platform. The data for the flag and probe tests were analyzing using a Genotype × Sex ANOVA, while the data from the acquisition phase was analyzed using a Genotype × Sex × Day ANOVA with repeated measures on the Day factor (four levels) and the data from the reversal test (conducted only between *Homer2^AA/AA^* and their WT mice) was analyzed using a Genotype × Sex × Trial ANOVA, with repeated measures on the Trial factor (four levels).

### Novel object reactivity test

*GRM5^AA/AA^* mice exhibit reduced signs of negative affect, relative to their WT controls to include lower novel object reactivity (Campbell et al., 2019). Thus, a novel object reactivity test was employed to compare genotypic differences in agoraphobic/neophobic behavior, as one behavioral index of anxiety-like behavior between *Homer2^AA/AA^* and WT mice. For comparison, novel object reactivity was determined in a separate study of WT and *GRM5^R/R^* mice. Mice were placed into a black, open field (45 × 45 × 45 cm) containing one small, inedible object for 2 min. During that time, animals were allowed to explore and interact with the object. The number of contacts were tracked by an experimenter blind to the genotype of the animals and the time in contact was recorded using a stopwatch (in seconds), as conducted in prior studies of *Homer2* null mutant mice (Szumlinski et al., 2005a, b). The data were analyzed using a Genotype × Sex ANOVA.

### Elevated plus maze

An elevated plus-maze procedure was also employed as an additional index of agoraphobic behavior. *Homer2^AA/AA^*, *GRM5^AA/AA^*, or *GRM5^R/R^* mice and their WT controls were placed on the center intersection of a four-arm radial plus maze with two white open arms and two black-walled arms 24 cm high. Each arm measured 123 cm long × 5 cm wide. The number of open-arm and closed-arm entries, and total time spent in an open arm were monitored for a 5-min trial by a trained observer who was blind to mouse genotype. The time spent in and entries into the open arms, as well as the total number of arm entries were analyzed using a Genotype × Sex ANOVA.

### Marble burying

The marble burying test was used to further compare neophobic behavior between *Homer2^AA/AA^* and their WT mice (Njung’e and Handley, 1991). The two lines of *GRM5* mutant mice were not available at the time of study and, thus, were not assayed for marble-burying. In our paradigm, 25 small black marbles (13 mm in diameter) were arranged in four rows of five marbles each in a standard Plexiglas rat cage (12 × 8 × 6 cm), lined (∼10 cm deep) with clean sawdust bedding. The latency to start burying the marbles was determined by a blind observer using a stopwatch, and the total number of marbles completely buried was recorded at the end of the 20-min session. The test cages were cleaned with disinfectant between animals and fresh bedding employed for each subject. The data were analyzing using a Genotype × Sex ANOVA.

### Light/dark shuttle box

The light/dark shuttle box test compared the photophobic/agoraphobic behavior between *Homer2^AA/AA^* and WT mice. The two lines of *GRM5* mutant mice were also not available at the time of study and, thus, were not assayed in this paradigm. Mice were placed into a polycarbonate box (46 cm long × 24 cm high × 22 cm wide) containing distinct open (light) and closed (dark) environments for a 15-min trial. These two environments were separated by a central divider with an opening. Mice were first placed on the dark side, and the latency to enter the light side, number of light-side entries, and total time spent in the light-side of the shuttle box were recorded using ANY-Maze tracking software (RRID:SCR_014289). An increase in latency to enter the light, uncovered, side was interpreted as an index of anxiety-like behavior. The data were analyzed using a Genotype × Sex ANOVA.

### Porsolt swim test

*GRM5^R/R^* mice exhibit less floating behavior in a forced swim test than their WT controls (Guo et al., 2016), suggesting that Homer2 hyperphosphorylation and disrupted mGlu5-Homer scaffolds favors an active coping strategy in response to a physical swim stressor. To test this hypothesis, *Homer2^AA/AA^* and WT mice were compared in a forced swim test in which each mouse was placed into an 11 cm diameter cylindrical container, filled with room-temperature water, and the latency to first exhibit immobility (defined as no horizontal or vertical displacement of the animal’s center of gravity for 5 s), total time spent immobile, and the numbers of immobile episodes were monitored throughout the entire 6-min trial period using ANY-Maze tracking software. For comparison, we also analyzed data obtained from an earlier forced swim study of WT and *GRM5^AA/AA^* mice, which employed a different swim test procedure that involved a larger diameter pool (30 cm), a longer duration of swimming (15 min) and the latency to first float, as well as the number of floating episodes, were recorded manually by a researcher blind to the genotype of the mice. In both studies, the data were analyzed using a Genotype × Sex ANOVA.

### Cocaine-induced place conditioning

The dose–response function for cocaine-induced place-conditioning is shifted markedly to the left in *GRM5^AA/AA^* mice, relative to WT controls, with *GRM5^AA/AA^* mice exhibiting a place-preference to 3 mg/kg cocaine but a very robust place-aversion to 10 and 30 mg/kg cocaine (Campbell et al., 2019). Thus, we compared the capacity of 3 versus 30 mg/kg cocaine to elicit place-conditioning between *Homer2^AA/AA^*, *GRM5^R/R^* mice and their WT controls. To induce place-conditioning, a two-compartment apparatus was employed in which the compartments were tactilely (floor texture) and visually (wall pattern) distinct. Behavior was recorded throughout the experiment using an ANY-Maze digital video-tracking system. Conditioning commenced with a 15-min habituation session to familiarize the animals to the entire apparatus. The next day served as a preconditioning test (Pre-Test) in which the animals again had 15 min of free-access to both compartments. Animals then received an intraperitoneal injection of saline (vol = 10 ml/kg) and were confined to one of the compartments for 15 min. The next day, mice were injected intraperitoneally with cocaine (3 or 30 mg/kg; Sigma) and then confined to the opposite compartment for 15 min. This conditioning procedure was repeated for a total of four conditioning sessions per side. Following the final conditioning session, animals were then allowed free-access to the entire apparatus in a drug-free state during a 15-min postconditioning test (Post-Test). Amount of time spent on the cocaine-paired versus saline-paired compartment during the Post-Test served to index cocaine reward/aversion and this CPP Score was analyzed using a Genotype × Sex × Dose ANOVA.

### Spontaneous and cocaine-induced locomotor activity

Although published comparisons of *GRM5^R/R^* versus *GRM5^AA/AA^* mice argue that merely perturbing mGlu5-Homer binding does not reliably affect the locomotor-activating or -sensitizing properties of cocaine (Park et al., 2013), Homer2 expression within both the prefrontal cortex (Ary et al., 2013) and in the nucleus accumbens (Szumlinski, 2004) actively regulates cocaine-induced locomotor activity. Thus, we compared the distanced traveled by *Homer2^AA/AA^* and WT mice during the first cocaine-conditioning session to index the acute locomotor response to cocaine, and the difference in the distance traveled from the first to fourth cocaine-conditioning session (δ Distance) indexed the development of locomotor sensitization. In addition to these measures of cocaine-induced locomotion, we also examined for genotypic differences in the distance traveled during the Pre-Test session to index behavioral reactivity to a novel environment, the distance traveled during the first saline-conditioning session to index spontaneous locomotor activity and the difference in the distance traveled from the first to fourth saline-conditioning session to index habituation to a neutral environment. All of these variables were analyzed using Genotype × Sex ANOVAs, with Cocaine Dose as an additional between-subjects factor, as appropriate.

### Cocaine-induced taste aversion

As an alternate index of cocaine aversion, a distinct cohort of *Homer2^AA/AA^*, *GRM5^AA/AA^*, and their WT mice were tested under cocaine-induced taste-aversion procedures. *GRM5^R/R^* mice were not available at the time of study. To induce a cocaine-conditioned taste-aversion, fluid access was scheduled for 1 h/d (Stafstrom-Davis et al., 2001; Kusuhara et al., 2013). For this, group-housed mice were placed into individual drinking cages in the colony room and allowed to habituate for 1 h. At the end of the 1 h habituation session, mice were presented with a sipper tube containing water and allowed to drink for 1 h at which time they were returned to their home cage. Over the next 3 d, mice underwent additional 1-h drinking sessions during which they were presented with a 10% sucrose (w/v) solution. Following the 3rd sucrose drinking session, all mice were injected IP with 30 mg/kg cocaine just before return to their home cage. The next day, mice were again offered the 10% sucrose solution in a taste-conditioning test. Fluid intake was recorded by bottle weight before and after the 1-h drinking sessions and intake calculated based on the body weight of the animal. The average sucrose intake on the days before cocaine injection were analyzed using a Genotype × Sex ANOVA, as was the difference in sucrose intake between the test and the baseline sucrose drinking sessions (δ Intake), which served to index taste-aversion.

### Cocaine-induced stereotypy

Higher cocaine doses induce both locomotor hyperactivity (measured as the distance traveled), as well as focused, stereotyped behaviors (Kuczenski et al., 1991), which are not reliably detected using digital-tracking software. Thus, to determine whether or not the effect of the *Homer2^AA/AA^* mutation on the acute aversive properties of cocaine might reflect an insensitivity to the stereotypy-inducing properties the drug, the same mice tested for cocaine-induced taste-aversion where then tested for cocaine-induced stereotypy, a day following the taste-conditioning test. For this, mice were injected IP with 30 mg/kg cocaine and placed into an activity chamber for 60 min. Every 10 min, the behavior of the mice was observed for 30 s and scored by a trained observer who was blind to the genotype of the mice using the following behavioral rating scale: 0 = asleep or still; 1= grooming or mild licking; 2 = continuous, exploratory locomotion along the horizontal plane for the entire 30 s without rearing; 3 = continuous, exploratory locomotion along the horizontal plane for the entire 30 s with rearing; 4 = bouts of locomotion along the horizontal plane/“darting” without rearing or sniffing; 5 = darting with bouts of rearing or sniffing; 6 = continuous sniffing for 30 s without horizontal locomotion or rearing; 7 = continuous sniffing for 30 s while rearing; 8 = patterned sniffing or head-bobbing in a fixed location for <30 s; 9 = patterned sniffing or head-bobbing in a fixed location for the entire 30 s; 10 = continuous gnawing or focused grooming; 11 = bizarre dyskinetic movements or seizures (adapted from Kalivas et al., 1988). The sum of the stereotypy scores over the 60-min period were analyzed using a Genotype × Sex ANOVA.

## Results

### Generation of phosphorylation site *Homer2* mutant KI mouse

To assess the *in vivo* roles of activity-regulated Homer2 phosphorylation and interactions with mGlu5 we created a Homer2 phosphorylation site mutant KI mouse using CRISPR/Cas9 technology that harbored alanine substitutions at S117 and S216 in Homer2 (*Homer2^AA/AA^*). Mice were viable and were born at expected Mendelian ratios.

Total levels of Homer2, Homer1, Homer1a and mGlu5 in cortical lysates of *Homer2^AA/AA^* mice were normal (Fig. 1*C*). To evaluate levels of phosphorylated S216 Homer2 in *Homer2^AA/AA^* mice, acute cortical slices were prepared and treated with bicuculline and 4-aminopyridine (Bic/4AP; 5 min) to induce neuronal activity. Levels of p(S216)-Homer2 were low in untreated slices and strongly induced with Bic/4AP treatment in wild-type (WT), but not Homer2*^AA/AA^* mice (Fig. 1*D*). Bic/4AP-induced phosphorylation of Homer1 at S117 was normal in slices from *Homer2^AA/AA^* mice.

### Preventing phosphorylation-dependent changes in Homer2-mGlu5 scaffolding does not alter sensorimotor processing or gating

**Figure 2.**
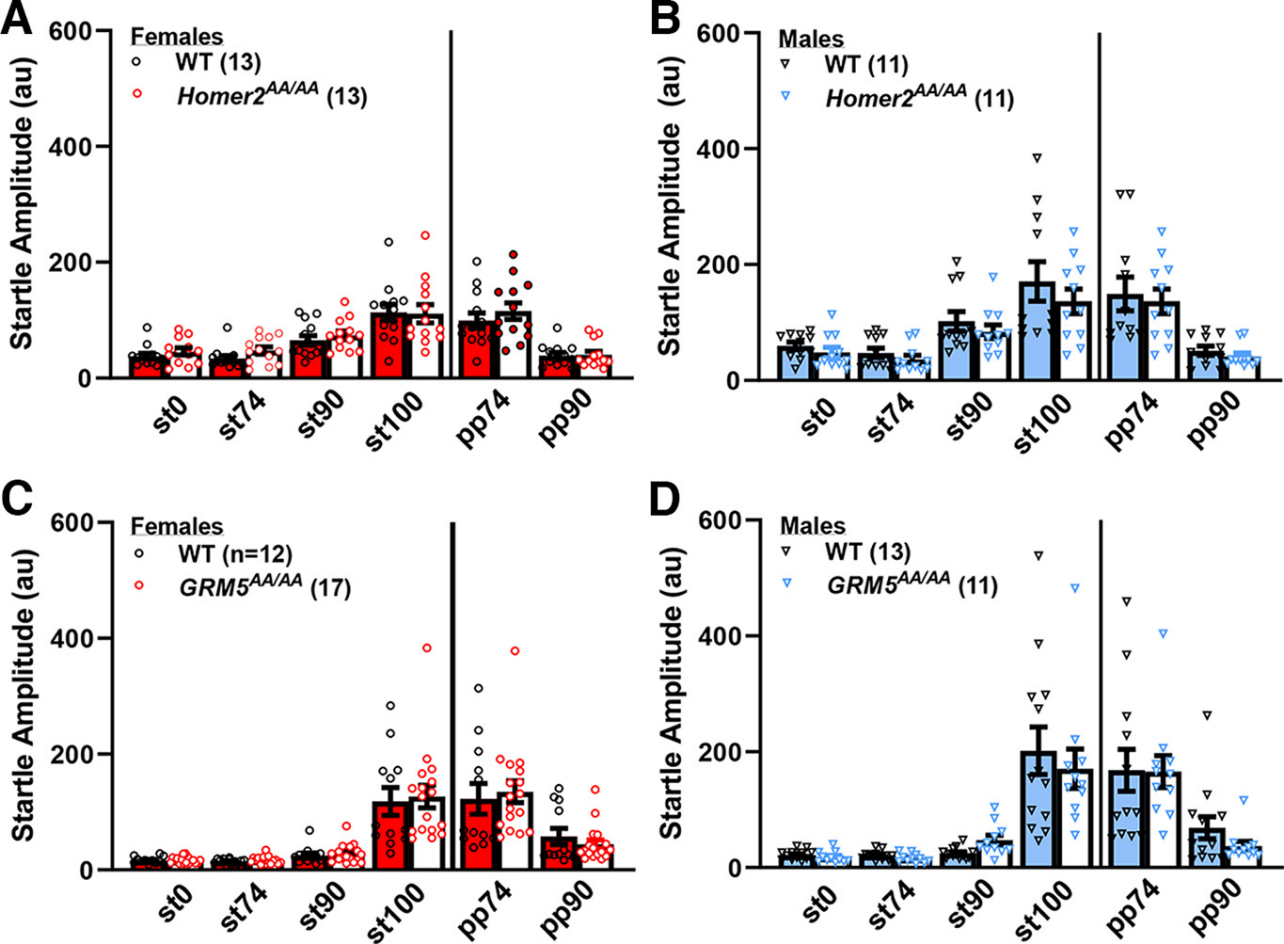
Phosphorylation site mutants of Homer2 or mGlu5 that prevent their dynamic regulation of interactions do not affect acoustic startle or PPI. Summary of the effects of the *Homer2^AA/AA^* (***A***, ***B***) and the *GRM5^AA/AA^* (***C***, ***D***) mutations on the magnitude of startle (expressed as arbitrary units or au) in response to different acoustic stimuli in the absence (st0–st100) and presence of a 74 and 90 dB prepulse (respectively, PPI74 and PPI90). The data for female mice are presented in red in the left panels, and the data for males are presented in blue in the right panels. The data represent the means ± SEMs of the number of mice indicated in parentheses.

When WT and *Homer2^AA/AA^* mice were tested for acoustic startle, we detected no difference in either startle magnitude (st0-st100) or PPI, although the females in this study exhibited lower startle magnitude in the absence of a prepulse, than their male counterparts (for acoustic stimuli, Stimulus effect: *F*_(3,132)_ = 49.08, *p* < 0.0001; Sex effect: *F*_(1,44)_ = 6.58, *p* = 0.01; no Genotype effect or interactions between factors, *p*s > 0.20; for PPI, Prepulse effect: *F*_(1,44)_ = 84.94, *p* < 0.0001; no Sex or Genotype effects or interactions, *p*s > 0.22; Fig. 2*A* vs *B*).

A very similar pattern of acoustic startle and PPI results were observed in a study of *GRM5^AA/AA^* mice and their WT controls. Irrespective of genotype, females (Fig. 2*C*) startled significantly less than males in the absence of a prepulse (Sex effect: *F*_(1,44)_ = 6.58, *p* = 0.01; Stimulus effect: *F*_(3,132)_ = 49.08, *p* < 0.0001; all other *p*s > 0.10; Fig. 2*D*), but no genotypic differences were detected for PPI (PrePulse effect: *F*_(1,44)_ = 84.94, *p* < 0.0001; no Sex or Genotype effects or interactions, *p*s > 0.23). Taken together, these data indicate that phospho-mutations that affect activity- (*Homer2^AA/AA^)* or signal-regulated (*GRM5^AA/AA^*) Homer2-mGlu5 binding do not alter sensorimotor processing or gating.

### Phospho-mutations affecting Homer2-mGlu5 scaffolds facilitate performance in the Morris water maze

To examine the effects of altering Homer2-mGlu5 scaffolding on spatial learning and memory, we compared *Homer2^AA/AA^*, *GRM5^AA/AA^*, and *GRM5^R/R^* mice to their respective WTs in a Morris water maze paradigm.

#### Maze acquisition

**Figure 3.**
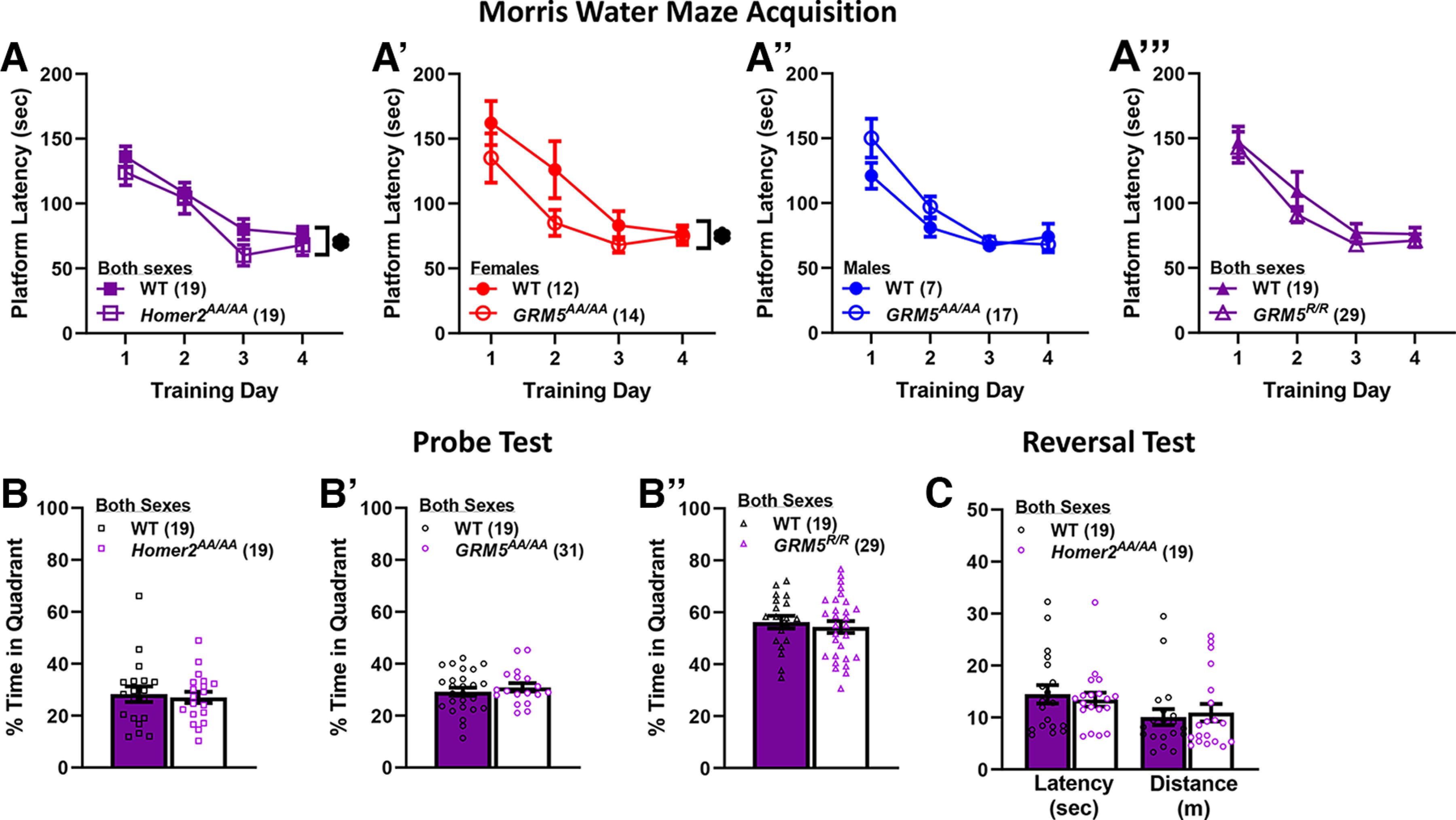
Phosphorylation site mutants of Homer2 or mGlu5 that prevent their dynamic regulation of interactions impair learning in a Morris water maze. Summary of the effects of the *Homer2^AA/AA^* (***A***), the *GRM5^AA/AA^* (***A’***, ***A’’***), and the *GRM5^R/R^* (***A’’’***) mutations on the time taken to locate a hidden platform in a Morris water maze task. As an interaction with the Sex factor was detected only in the study of *GRM5^AA/AA^* mice, the data are presented separately for females (red) and males (blue), while the data are collapsed across sex for the other mutants. ***B–B’’***, Summary of the results of the memory probe test for *Homer2^AA/AA^*, *GRM5^AA/AA^*, and *GRM5^R/R^* studies, respectively, indicating no effects of the mutations on recall. ***C***, Comparison of the total latency and distance traveled during a four-trial reversal learning test between *Homer2^AA/AA^* mice and their WT controls, indicating no difference in reversal learning. The data represent the means ± SEMs of the number of mice indicated in parentheses. *p < 0.05 for a main Genotype effect.

Overall, the latency to locate the hidden platform during the acquisition phase of Morris water maze training was shorter in *Homer2^AA/AA^* versus WT mice (Genotype effect: *F*_(1,34)_ = 5.71, *p* = 0.02), although both genotypes exhibited an equivalent rate of learning over the four training trials (Session effect: *F*_(3,102)_ = 15.22, *p* < 0.0001; Session × Genotype: *p* = 0.41; Fig. 3*A*). There were no sex differences detected for the rate of maze learning in WT or *Homer2^AA/AA^* mice (Sex effect and interactions, *p*s > 0.08).

In contrast to *Homer2^AA/AA^* mice (Fig. 3*A*), the genotypic difference in Morris maze learning was sex-dependent in the study of *GRM5^AA/AA^* mice (Genotype × Sex: *F*_(1,46)_ = 5.31, *p* = 0.03), with female *GRM5^AA/AA^* mice exhibiting a shorter mean latency to locate the platform versus their female WT controls (*t*_(24)_ = 2.09, *p* = 0.047; Fig. 3*A****’***). No significant genotypic difference was apparent between male WT and *GRM5^AA/AA^* mice (*t*_(22)_ = 1.19, *p* = 0.25; Fig. 3*A’’*). However, akin to *Homer2^AA/AA^* mice, the *GRM5^AA/AA^* mutation did not significantly affect the rate of learning across the four training sessions (Session effect: *F*_(1,138)_ = 29.03, *p* < 0.0001; Session interactions, all *p*s > 0.20).

In contrast to the phospho-mutant mice, we detected no group differences in Morris maze acquisition between WT and *GRM5^R/R^* mice that cannot bind Homer (Session effect: *F*_(3,138)_ = 29.03, *p* < 0.001; all other *p*s > 0.12; Fig. 3*A’’’*). Taken together, these data indicate that mutations that alter activity-regulated Homer-mGlu5 binding facilitate performance on the Morris maze, while that which disrupts Homer-mGlu5 binding has no effect.

#### Probe test for spatial memory

To index spatial memory, we removed the platform and compared the relative time spent by mice in the former platform quadrant versus the opposite quadrant as an index of spatial recall (Stavnezer et al., 2002). No genotypic differences in this measure of recall were detected in the study of *Homer2^AA/AA^* mice (Genotype × Sex ANOVA, all *p*s > 0.56; Fig. 3*B*), *GRM5^AA/AA^* (Genotype × Sex ANOVA, all *p*s > 0.62; Fig. 3*B’*), or *GRM5^R/R^* mice (Genotype × Sex ANOVA, all *p*s > 0.27; Fig. 3*B’’*). Further, an examination of data for alternate indices of spatial memory (e.g., latency to first visit the former platform location etc.) also failed to indicate any genotypic differences in spatial recall in the studies of *Homer2^AA/AA^* or *GRM5^R/R^* mice (Table 1). Further, the *Homer2^AA/AA^* mutation did not affect the acquisition of a new platform location during reversal training (Genotype × Sex × Trial ANOVA for the latency to locate platform, Trial effect: *F*_(3,102)_ = 61.92, *p* < 0.0001; all other *p*s > 0.40; Fig. 3*C*).

**Table 1:** Summary of the descriptive statistics and results of the Genotype × Sex ANOVAs conducted on the data from the alternate indices of spatial memory recall obtained during Morris water maze testing of *Homer2^AA/AA^*, *GRM5^AA/AA^*, and *GRM5^R/R^* mutants and their WT mice

Study:	*Homer2^AA/AA^*		*GRM5^AA/AA^*		*GRM5^R/R^*	
Genotype:	WT(*n* = 19)(10 F, 9 M)	Mutant(*n* = 19)(10 F, 9 M)	WT(*n* = 19)(12 F, 7 M)	Mutant(*n* = 31)(17 F, 14 M)	WT(*n* = 19)(12 F, 7 M)	Mutant(*n* = 29)(13 F, 16 M)
Latency to enter former platform location (s)	15.4 ± 4.2	11.5 ± 2.8	15.9 ± 2.1	10.8 ± 1.3	12.3 ± 3.2	12.6 ± 2.7
	All *p*s > 0.45		Genotype: *p* = 0.10 Sex: *F*_(1,49)_ = 7.64, *p* = 0.008 Interaction: *p* = 0.46		All *p*s > 0.64	
Time in former quadrant (s)	24.0 ± 2.6	23.8 ± 1.9	24.5 ± 1.6	26.4 ± 0.9	24.0 ± 1.3	24.0 ± 1.0
	All *p*s > 0.50		All *p*s > 0.13		All *p*s > 0.13	
Number of former platform location entries	5.1 ± 0.7	5.9 ± 0.7	8.2 ± 0.8	11.0 ± 0.7	7.9 ± 1.2	9.6 ± 1.1
	All *p*s > 0.15		Genotype: *F*_(1,49)_ = 5.62, *p* = 0.02 Other *p*s > 0.49		All *p*s > 0.35	
Total distance (m)	16.0 ± 1.0	16.2 ± 1.5	13.0 ± 1.0	13.5 ± 0.7	16.6 ± 1.1	16.9 ± 0.8
	Sex: *F*_(1,37)_ = 6.40, *p* = 0.02 Other *p*s > 0.55		All *p*s > 0.27		All *p*s > 0.27	

The data represent the means ± SEMs of the number of mice indicated in parentheses. The number of female (F) and male (M) mice for each genotype is also indicated in parentheses. *p < 0.05 versus WT.

Interestingly, although *GRM5^AA/AA^* mice did not differ from their WT controls with respect to the relative time spent in the former platform quadrant (Genotype × Sex ANOVA, all *p*s > 0.10; Fig. 3*B’*), they entered the former platform location significantly more times during the probe test than did their WT controls (Table 1), indicative of better recall. The *GRM5^AA/AA^* mutation not only reduces mGlu5-Homer binding avidity, but also perturbs BDNF-induced or dopamine-induced, mGlu5-mediated potentiation of NMDA receptor currents (Park et al., 2013). This collection of Morris water maze findings suggest that enhancing recall via preventing mGlu5 phosphorlyation occurs independent of mGlu5-Homer2 interactions.

### Phospho-mutations affecting Homer2-mGlu5 scaffolds reduce signs of negative affect

*GRM5^R/R^* and *Fmr1* KO mice, both of which have disrupted mGlu5-Homer scaffolds, exhibit hypoanxiety under various paradigms that assay negative affect (Z.H. Liu and Smith, 2009; Guo et al., 2016). To test the hypothesis that regulated interactions of mGlu5 or Homer2 contribute to negative affect, we characterized the affective phenotype of both *GRM5^AA/AA^* and *Homer2^AA/AA^* mice, as well as expanded on the published hypoanxious phenotype of *GRM5^R/R^* mice (Guo et al., 2016).

#### Elevated plus maze

**Figure 4.**
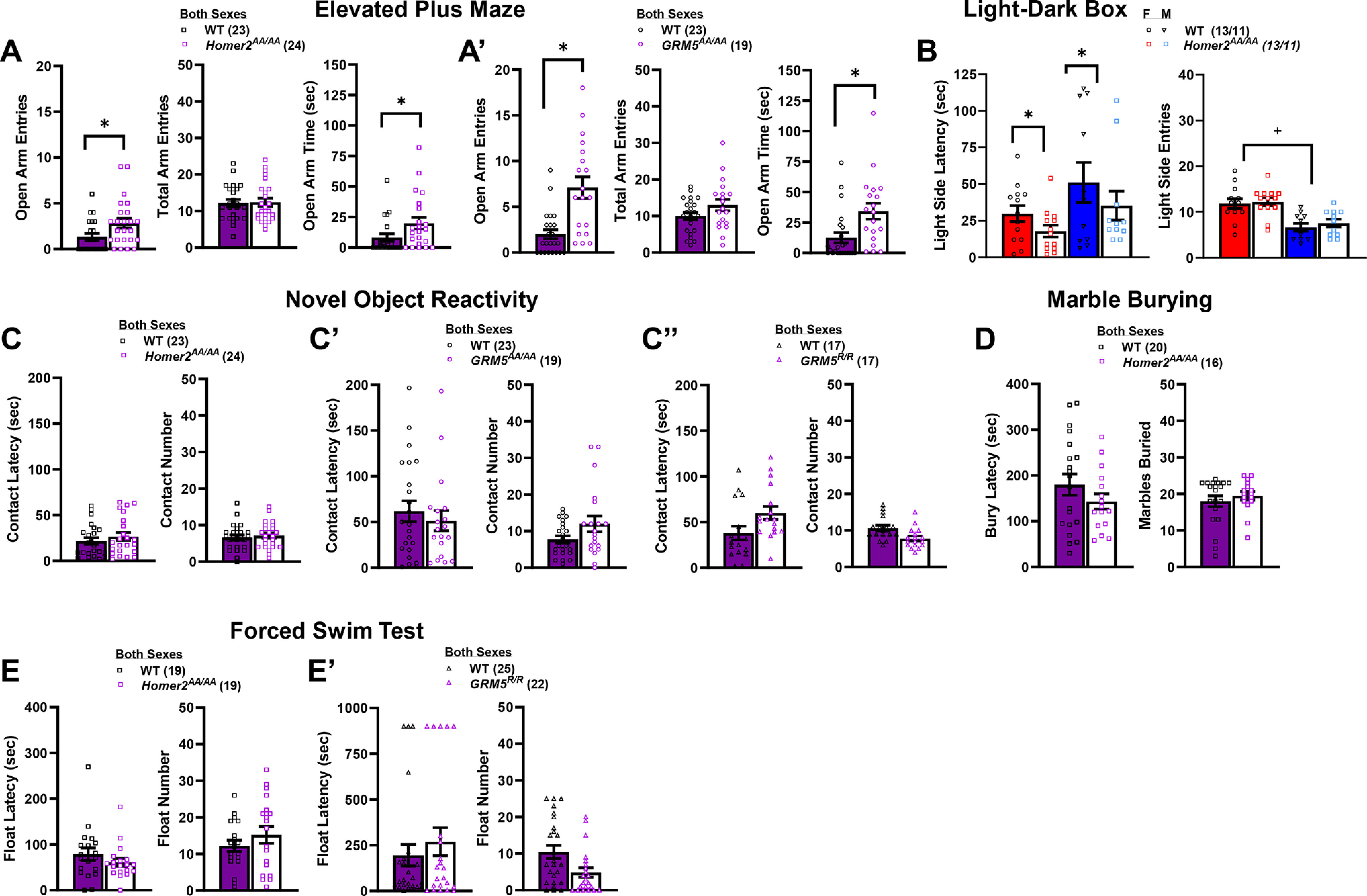
Phosphorylation site mutants of Homer2 or mGlu5 that prevent their dynamic regulation of interactions reduce some signs of negative affect. Summary of the effects of the *Homer2^AA/AA^* (***A***) and the *GRM5^AA/AA^* (***A’***) mutations on behavior in an elevated plus maze assay, and the effects of the *Homer2^AA/AA^* mutation on behavior in a light-dark shuttle-box (***B***), indicating less anxiety-like behavior in the mutant mice. Note that because of a sex difference, the data for the light-dark shuttle-box are presented separately for females (red) and males (blue). ***C’–C’’***, Summary of the results of a novel object reactivity test for *Homer2^AA/AA^*, *GRM5^AA/AA^*, and *GRM5^R/R^* mutations, respectively, indicating increased anxiety-like behavior in *GRM5^R/R^* mutants only. ***D***, Comparison of behavior expressed by *Homer2^AA/AA^* mice and their WT controls in the marble-burying test, indicating no difference in this assay. ***E***, ***E’***, Summary of the results of a forced swim test for *Homer2^AA/AA^* (8 min) and *GRM5^AA/AA^* mutations (15 min), respectively, indicating less passive coping behavior in *GRM5^AA/AA^* mutants. The data represent the means ± SEMs of the number of mice indicated in parentheses. *p < 0.05 for a main Genotype effect, +*p* < 0.05 for a main Sex effect.

Both *GRM5^R/R^* and *Fmr1* KO mice with disrupted scaffolding exhibit low levels of anxiety-like behavior in the elevated plus maze (Guo et al., 2016), posing intact mGlu5-Homer2 scaffolds as drivers of negative affect in this paradigm. However, preventing phosphorylation-dependent mGlu5-Homer2 uncoupling also increased both the number of open arm entries (Genotype effect: *F*_(1,49)_ = 5.04, *p* = 0.03), and the time spent in the open arm (*F*_(1,49)_ = 4.19, *p* = 0.05), without affecting the total number of arm entries (Genotype effect, *p* = 0.44; Fig. 4*A*). Although females exhibited more open and total arm entries than males (for open entries: Sex effect: *F*_(1,49)_ = 5.53, *p* = 0.02; for total entries: Sex effect: *F*_(1,49)_ = 10.64, *p* = 0.002), the anxiolytic effect of the *Homer2^AA/AA^* mutation did not vary as a function of sex (Genotype × Sex interaction: for open arm entries, *p* = 0.64; for open arm time, *p* = 0.81; data not shown).

Curiously, *GRM5^AA/AA^* mice exhibited a very similar anxiolytic profile as *Homer2^AA/AA^* mice, with phospho-mutants entering the open arms more often (Genotype effect: *F*_(1,41)_ = 15.33, *p* < 0.0001) and spending a longer time in the open arms than their WT controls (Genotype effect: *F*_(1,41)_ = 7.10, *p* = 0.01). The effect of the *GRM5^AA/AA^* deletion was independent of sex (for both variables, Sex effect and interactions, *p*s > 0.39) and did not generalize to total arm entries (Genotype × Sex ANOVA, all *p*s > 0.13; Fig. 4*A’*). Thus, phospho-mutations impede dynamic regulation of mGlu5 and its binding partners, with either Pin1 (*GRM5^AA/AA^*) or Homer2 (*Homer2^AA/AA^*) and produce a similar phenotype as that observed in *GRM5^R/R^* mice that cannot bind Homer, suggesting that regulation of mGlu5-Homer scaffolds promotes anxiety.

#### Light-dark shuttle box

As measure of photophobia/agoraphobia, we also compared *Homer2^AA/AA^* and their WT controls in a light-dark shuttle-box assay. Consistent with their anxiolytic phenotype in the elevated plus maze (Fig. 4*A*), *Homer2^AA/AA^* mice exhibited a shorter latency to first enter the light-side of a light-dark shuttle box (Genotype effect: *F*_(1,47)_ = 6.32, *p* = 0.02; Fig. 4*B*, left). Although, the females in this study expressed less anxiety-like behavior overall (Sex effect: *F*_(1,47)_ = 4.70, *p* = 0.04), the genotypic difference in this measure was sex-independent (Genotype × Sex: *p* = 0.57). Females also exhibited approximately twice as many light-side entries as did males (Sex effect: *F*_(1,47)_ = 28.44, *p* < 0.0001; Fig. 4*B*, right); however, the *Homer2^AA/AA^* mutation did not affect the number of light-side entries in either sex (no Genotype effect or interaction, *p*s > 0.45). *GRM5^AA/AA^* and *GRM5^R/R^* mice were not available at the time of study and, thus, were not assayed for photophobia.

#### Novel object reactivity

As a measure of neophobia and an alternate measure of agoraphobia, we also compared *Homer2^AA/AA^*, *GRM5^AA/AA^* and *GRM5^R/R^* mice to their respective WTs in a novel object reactivity paradigm. In contrast to the prior two assays, we failed to detect an effect of the *Homer2^AA/AA^* mutation on the latency to first contact the novel object (Genotype × Sex ANOVA, all *p*s > 0.20) or the number of novel object contacts (Genotype × Sex ANOVA, all *p*s > 0.10; Fig. 4*C*). Likewise, there was no effect of the *GRM5^AA/AA^* mutation on these measures (Genotype × Sex ANOVAs, for latency: all *p*s > 0.12; for contact number, all *p*s > 0.10; Fig. 4*C’*). However, *GRM5^R/R^* mice exhibited a longer latency to first contact the novel object (Genotype effect: *F*_(1,33)_ = 4.15, *p* = 0.03; Sex effect and interaction, *p*s > 0.58; Fig. 4*C’’*), and made fewer contacts with the novel object (Genotype effect: *F*_(1,33)_ = 4.28, *p* = 0.03; Sex effect and interaction, *p*s > 0.12), indicating that absolute disruption of mGlu5-Homer binding increases anxiety-like behavior in this assay.

#### Marble burying

As an alternate index of neophobia, *Homer2^AA/AA^* and WT mice were also compared in a marble-burying test. Although, *Homer2^AA/AA^* mice tended to exhibit a shorter latency to bury and to bury fewer marbles than their WT controls, no significant sex or genotypic differences were detected for either variable (Genotype × Sex ANOVAs: for latency, all *p*s > 0.29; for number buried, all *p*s > 0.34; Fig. 4*D*). *GRM5^AA/AA^* and *GRM5^R/R^* mice were not available at the time of study and, thus, were not assayed for marble-burying.

#### Forced swim test

Both *Fmr1* KO and *GRM5^R/R^* mice exhibit less floating behavior in the forced swim test, relative to their WT controls (Uutela et al., 2014; Guo et al., 2016), indicating an important role for mGlu5-Homer binding in regulating behavioral reactivity to a swim stressor. In contrast, no differences were observed between male and female *Homer2^AA/AA^* and WT mice regarding the latency to first float or the number of floats in a forced swim test (Genotype × Sex ANOVAs, for latency, all *p*s > 0.25; for floating episodes, all *p*s > 0.20; Fig. 4*E*). The latency to first float exhibited by both male and female WT and *GRM5^AA/AA^* mice was longer than that exhibited by *Homer2^AA/AA^* mice, which likely related to the larger diameter pool employed in this study. However and regardless of sex, *GRM5^AA/AA^* mice did not differ from their WT controls in terms of their latency to first float (Genotype × Sex ANOVA, all *p*s > 0.11; Fig. 4*E’*), although they exhibited fewer floating episodes (Genotype effect: *F*_(1,47)_ = 5.92, *p* = 0.02; Sex effect and interaction, *p*s > 0.75) in a manner akin to *GRM5^R/R^* mice (Guo et al., 2016). These forced swim data indicate that mutations affecting dynamic regulation of mGlu5-Homer scaffolding do not impact behavioral reactivity to a swim stressor, while those that reduce scaffolding increase active coping behavior.

### Phospho-mutation of Homer2 reduces the aversive properties of cocaine, without impacting cocaine-induced psychomotor activation

#### Spontaneous and cocaine-induced locomotion

Constitutive *Homer2* deletion markedly increases sensitivity to the acute psychomotor-activating properties of cocaine, without altering spontaneous motor activity (Szumlinski et al., 2004). Similarly, *GRM5^AA/AA^* transgenic mice with deficits in dopamine/BDNF-regulated mGlu5 interactions also exhibit a greater locomotor response to acute cocaine, but fail to develop cocaine-induced locomotor sensitization, presumably because of their “presensitized” state (Park et al., 2013). In contrast, *GRM5^R/R^* mice with disrupted mGlu5-Homer interactions do not differ from WT controls with respect to cocaine-induced locomotion or locomotor sensitization (Park et al., 2013). To determine more specifically how manipulations of mGlu5-Homer2 binding impact psychomotor activity, we next examined for the effect of the *Homer2^AA/AA^* mutation on spontaneous and cocaine-induced changes in locomotor activity during place-conditioning procedures. Overall, females exhibited more novelty-induced locomotor hyperactivity than males during the Pre-Test session (females: 31.92 ± 1.43 m vs males: 27.48 ± 1.51 m), and mice injected with 30 mg/kg cocaine exhibited a greater conditioned locomotor response during the Post-Test session than those conditioned with 10 mg/kg cocaine, but there were no genotypic differences on any measures of spontaneous or cocaine-conditioned locomotor activity (for details, see Table 2).

**Table 2:** Summary of the descriptive statistics and results of the Genotype × Sex ANOVAs for data pertaining to the spontaneous locomotor activity of *Homer2^AA/AA^* and their WT mice

Variable	WT(*n* = 19)	*Homer2^AA/AA^*(*n* = 19)
Distance traveled during Habituation (m)	31.5 ± 1.6	28.0 ± 1.4
	Sex: *F*_(1,79)_ = 4.17, *p* = 0.045 All *p*s > 0.10	
Distance traveled following acute saline injection (m)	15.1 ± 1.4	16.7 ± 3.8
	All *p*s > 0.18	
Change in distance travelled from saline injection 1 to 4 (m)	0.7 ± 1.4	−0.15 ± 3.7
	All *p*s > 0.34	

The data represent the means ± SEMs of the number of mice indicated in parentheses.

**Figure 5.**
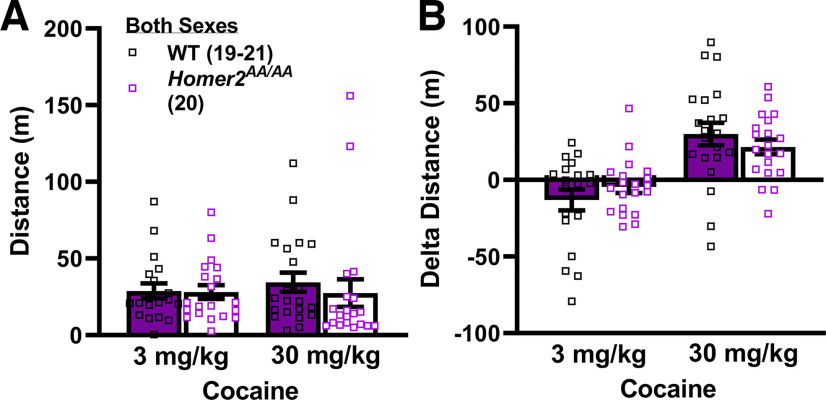
Phosphorylation site mutants of Homer2 that prevent activity-regulated interactions with mGlu5 exhibit normal locomotor responses to cocaine. Summary of the effects of the *Homer2^AA/AA^* mutation on the locomotor response to acute injection with either 3 or 30 mg/kg cocaine (***A***) and its change with repeated (4×) treatment (i.e., δ Distance; ***B***). The data represent the means ± SEMs of the number of mice indicated in parentheses.

No sex differences were observed for cocaine-induced changes in locomotor activity (Genotype × Sex × Dose ANOVA, all *p*s > 0.07). Thus, the data were collapsed across sex for re-analysis. Homer2 phospho-mutation did not alter the acute locomotor response to cocaine during the first place-conditioning session (Genotype × Dose ANOVA, *p*s > 0.60; Fig. 5*A*), nor did it alter the sensitization of locomotor activity elicited by repeated cocaine treatment, as defined by the increase in locomotor activity from the first to the fourth conditioning session (Dose effect: *F*_(1,79)_ = 37.6, *p* < 0.0001; Genotype effect and interaction, *p*s > 0.60; Fig. 5*B*). Thus, disrupting CAMKII-dependent phosphorylation of Homer2 does not alter cocaine-induced psychomotor activity nor its sensitization with repeated administration.

#### Cocaine place conditioning

**Figure 6.**
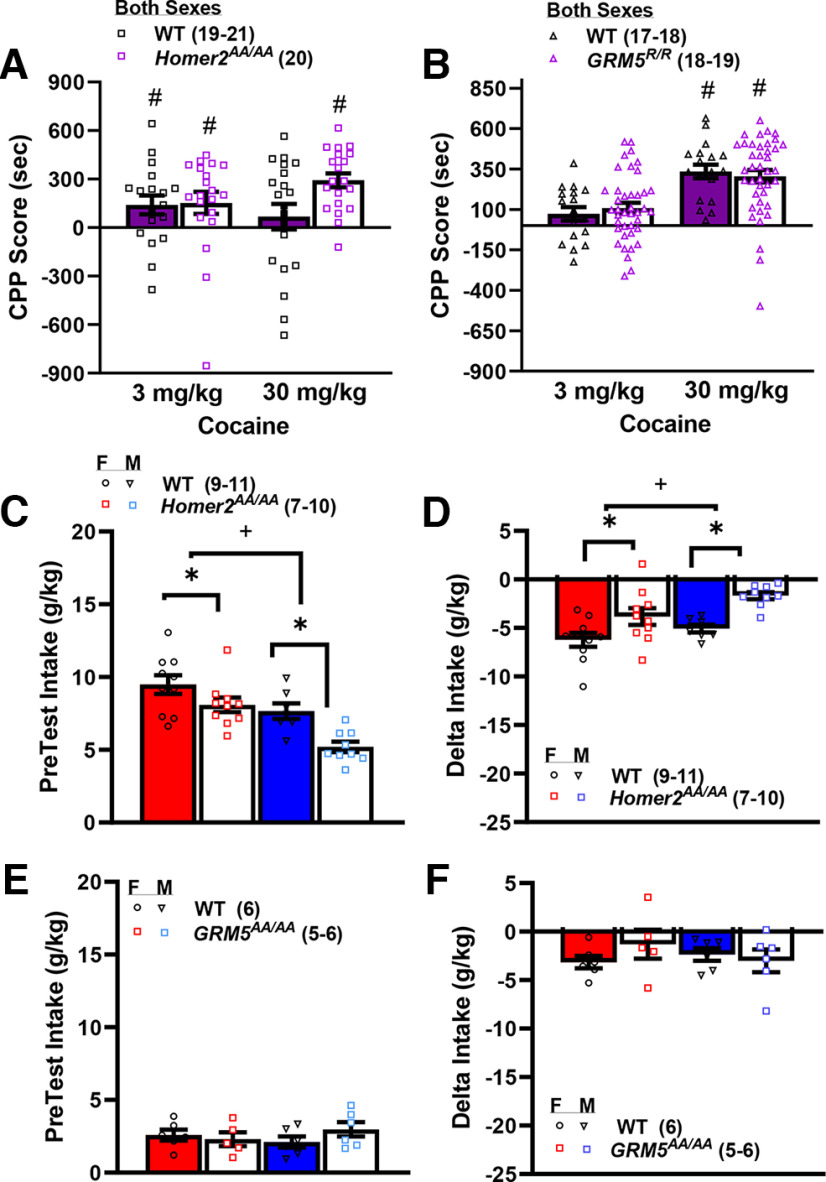
Phosphorylation site mutants of Homer2 that prevent activity-regulated interactions with mGlu5 reduces the aversiveness of high-dose cocaine. Summary of the effects of the *Homer2^AA/AA^* (***A***) and the *GRM5^R/R^* (***B***) mutation on the expression of place-conditioning elicited by four pairings of 3 or 30 mg/kg cocaine, indicating greater place-preference in *Homer2^AA/AA^* mutants at the higher cocaine dose. For panels ***A***, ***B***, #*p* < 0.05 versus 0 CPP Score (i.e., place-conditioning). Summary of the effects of the *Homer2^AA/AA^* mutation on baseline sucrose intake (***C***) and the change in sucrose intake induced by 30 mg/kg cocaine (i.e., δ Intake; ***D***), indicating lower sucrose intake but blunted conditioned taste aversion in mutant mice. ***E***, ***F***, Comparable taste-conditioning data for *GRM5^AA/AA^* mice indicating no effect of this mutation on sucrose intake or conditioned taste aversion. For panels ***C***, ***D***, *p < 0.05 main Genotype effect; +*p* < 0.05 main Sex effect; #*p* < 0.05 vs. 0 (one-sample *t*-test). The data represent the means ± SEMs of the number of mice indicated in parentheses.

*GRM5^AA/AA^* mice exhibit a very marked shift to the left in the dose–response function for cocaine-induced place-conditioning, with mutant mice exhibiting robust conditioned place-aversion to doses that elicit a place-preference in WT controls (Campbell et al., 2019). Herein, *Homer2^AA/AA^* mice exhibited what appeared to be an opposite phenotype (Fig. 6*A*), with both male and female mutant mice exhibiting more robust place-conditioning than WT controls at 30 mg/kg cocaine (Genotype × Side: *F*_(1,75)_ = 8.46, *p* = 0.005; Genotype × Dose: *p* = 0.09; all other *p*s > 0.17). Collapsing the data across sex, one-sample *t* tests (comparator = 0 s or no conditioning) were conducted independently for each genotype at each cocaine dose to confirm the presence of a conditioned response. As illustrated (Fig. 6*A*), 3 mg/kg cocaine elicited a significant place-preference in both WT and *Homer2^AA/AA^* mice and the magnitude of the place-preference was comparable (for WT, *t*_(17)_ = 2.38, *p* = 0.03; for *Homer2^AA/AA^*, *t*_(19)_ = 2.25, *p* = 0.04). In contrast, the 30 mg/kg cocaine dose elicited a significant place-preference only in the *Homer2^AA/AA^* mice (for WT, *t*_(19)_ = 0.85, *p* = 0.40; for *Homer2^AA/AA^*, *t*_(19)_ = 6.87, *p* < 0.0001). As illustrated in Figure 6*A*, place-ambivalence exhibited by the WT mice injected with 30 mg/kg cocaine in this study could be attributed to the relatively high degree of variability in the conditioned response; ∼1/3rd of WT mice tested at this dose exhibited a conditioned place-aversion (i.e., negative CPP score), whereas this dose elicited a place-aversion in only 1 *Homer2^AA/AA^* mouse and the magnitude of that aversion was less than that exhibited by the majority of cocaine-averse WT animals. While the place-conditioning data from studies of *GRM5^AA/AA^* (Campbell et al., 2019) versus *Homer2^AA/AA^* mice (Fig. 6*A*) argue that mGlu5-Homer2 interactions bidirectionally gate the motivational valence of high-dose cocaine, a comparable study of *GRM5^R/R^* mutants with an absolute disruption of mGlu5-Homer binding failed to detect any genotypic differences in place-conditioning, with neither WT nor mutant mice exhibiting conditioned aversion at the doses tested (Dose effect: *F*_(2,105)_ = 15.65, *p* < 0.0001; Sex effect: *p* = 0.08; other *p*s > 0.29; Fig. 6*B*), with one-sample *t* tests indicated trends toward a place-preference in both WT and *GRM5^R/R^* mice conditioned with 3 mg/kg cocaine (for WT: *t*_(16)_ = 1.75, *p* = 0.09; *GRM5^R/R^*: *t*_(18)_ = 2.03, *p* = 0.06) and a robust place-preference at 30 mg/kg cocaine (for WT, *t*_(17)_ = 7.91, *p* < 0.0001; for *GRM5^R/R^*: *t*_(18)_ = 5.49, *p* < 0.0001).

#### Cocaine taste conditioning

We next determined whether or not the genotypic differences in cocaine-induced place-conditioning exhibited by *Homer2^AA/AA^* (Fig. 6*A*) versus *GRM5^AA/AA^* mice (Campbell et al., 2019) extended to an assay of the acute aversive properties of the drug using taste-conditioning procedures. Before posttreatment with 30 mg/kg cocaine, fluid-restricted *Homer2^AA/AA^* mutants and males consumed significantly less of the 10% sucrose solution on average than did WT mice (Genotype effect: *F*_(1,35)_ = 12.94, *p* = 0.001; Sex effect: *F*_(1,35)_ = 19.42, *p* < 0.0001; interaction: *p* = 0.33; Fig. 6*C*). However, despite exhibiting lower baseline sucrose intake, both male and female *Homer2^AA/AA^* mice exhibited a smaller cocaine-conditioned taste aversion than their WT controls (Genotype effect: *F*_(1,35)_ = 18.29, *p* < 0.0001; Sex effect: *F*_(1,35)_ = 6.00, *p* = 0.02; interaction: *p* = 0.46; Fig. 6*D*). In contrast, *GRM5^AA/AA^* mice did not differ from their WT controls with respect to baseline sucrose intake (Genotype × Sex ANOVA, *p*s > 0.19; Fig. 6*E*) or the magnitude of the taste aversion (Genotype × Sex ANOVA, *p*s > 0.23; Fig. 6*F*).

#### Cocaine-induced stereotypy

We next related the apparent insensitivity of *Homer2^AA/AA^* mice to the aversive properties of high-dose cocaine to the magnitude of high-dose cocaine-induced stereotypy. For this, the same mice were injected with 30 mg/kg cocaine and stereotypy was recorded in an activity arena every 10 min for 1 h. No sex or genotypic differences were detected for the total stereotypy scores over the testing period (Genotype × Sex ANOVA, all *p*s > 0.20; WT = 13.47 ± 1.0; *Homer2^AA/AA^*=14.68 ± 0.48). Further there was no correlation between the magnitude of cocaine-induced taste aversion and the expression of stereotypy (*r* = −0.06; *p* = 0.74). When combined with the results for locomotor activity (Fig. 5), these stereotypy findings argue that the relative insensitivity of *Homer2^AA/AA^* mice to the aversive properties of cocaine does not reflect an effect of the mutation on cocaine-induced psychomotor activation.

### Cocaine induces a rapid disruption of striatal mGlu5-Homer that requires Homer2 phosphorylation

**Figure 7.**
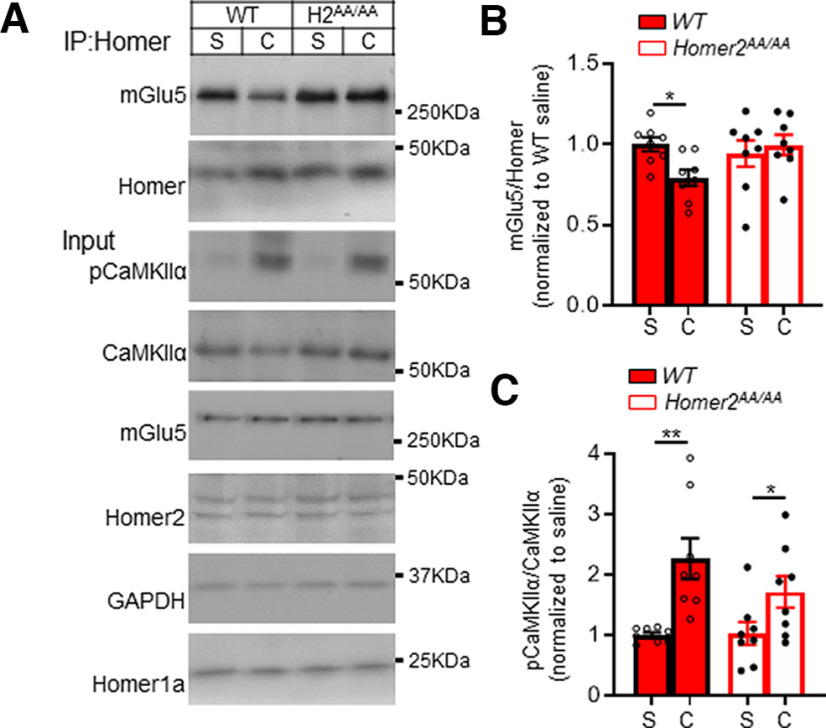
High-dose cocaine decreases mGluR5-Homer interactions in striatal lysates and is deficient in *Homer^2AA/AA^* mice. ***A***, Representative Western blottings from striatal lysates from WT or *Homer2^AA/AA^* mice (H2^AA/AA^) 1 h following acute treatment with saline (S) or cocaine (C; 20 mg/kg, i.p.). ***B***, Quantified group data of mGlu5 that co-immunoprecipitated with a pan-Homer antibody. Cocaine reduced the mGlu5-Homer co-IP in striatum of WT, but not *Homer2^AA/AA^* mice. *n* = 8 mice/group. Error bars represent SEM, ∗*p* < 0.05; Sidak’s multiple comparison test. ***C***, Quantified group data of the ratio of phosphorylated (P) T286 and active CaMKIIα to total CaMKIIα show that cocaine induced robust activation of CaMKIIα in the striatum of both WT and *Homer2^AA/AA^* mice; *n* = 8 mice/group. **p < 0.01, ∗*p* < 0.05; Sidak’s multiple comparison test. There is no change in the total levels of mGlu5, Homer2, Homer1a, CaMKIIa, and GAPDH in WT and *Homer2^AA/AA^* mice striatal homogenates with/without 1-h cocaine administration.

*Homer2^AA/AA^* mice show normal cocaine-conditioned reward in response to lower cocaine doses (≤10 mg/kg), but exhibit conditioned-reward at higher doses that are aversive to WT mice. These results suggest that high-dose cocaine stimulates phosphorylation of Homer2 at CaMKIIα sites, which causes a reduction in Homer-mGlu5 interactions. To test this possibility, WT or *Homer2^AA/AA^* mice were treated with a high cocaine dose (20 mg/kg, i.p.) and after 1 h, whole striatum was dissected and a co-immunoprecipitation (co-IP) of Homer and mGlu5 was performed. Cocaine treatment resulted in a decrease in mGlu5 co-IP with Homer in striatum of WT, but not *Homer2^AA/AA^*, mice (Fig. 7*A*,*B*). Cocaine also resulted in activation of CaMKIIα, as measured by p(T286)-CaMKIIα in striatal lysates (Fig. 7*C*). p(S216)-Homer2 was unable to be quantified in striatal lysates, in contrast to cortical lysates (Fig. 1*D*), because of the presence of a nonspecific band (data not shown).

## Discussion

The capacity of mGlu5 to bind the postsynaptic density scaffolding protein Homer2 has been implicated in gating the psychomotor-activating, rewarding and reinforcing properties of cocaine for over two decades (Swanson et al., 2001; Ghasemzadeh et al., 2003; Szumlinski et al., 2004; Knackstedt et al., 2010; Ary et al., 2013; Park et al., 2013; Loweth et al., 2014; Gould et al., 2015). In addition to a conserved CaMKIIα-directed phosphorylation site at S117, Homer2 also contains an additional phosphorylation site at S216 within its hinge region between the EVH1 domain that enables binding to interactors and its coiled-coil domain that allows for tetramerization with other Homer proteins to form multiprotein receptor scaffolds (Mizutani et al., 2008; Guo et al., 2015) CaMKIIα-mediated phosphorylation of these sites rapidly reduces Homer2 binding to mGlu5, as well as other interactors and this dynamical, activity-dependent, dissociation of Homer2-mGlu5 interactions is implicated in the behavioral and neuropathological phenotype of *Fmr1* KO mouse model of Fragile X syndrome (Guo et al., 2015). Here, we developed a *Homer2* KI mouse incapable of activity-dependent, CaMKIIα-mediated, phosphorylation of S117 and S216 to probe its relevance for striatal mGlu5 binding, sensorimotor, affective, and cognitive processing, as well as behavioral sensitivity to cocaine. We show that acute cocaine dissociates mGlu5-Homer2 complexes within striatum which requires Homer2 phosphorylation (Fig. 7). As summarized in Table 3, the spontaneous behavior of phospho-mutants incapable of actively-dependent dissociation of Homer2 scaffolds deviated little from their WT littermates, with *Homer2^AA/AA^* mutants exhibiting WT-levels of acoustic startle, prepulse inhibition of acoustic startle, spatial learning, memory and reversal learning in a Morris water maze task, anxiety-like behavior in novel object reactivity, marble-burying and forced swim tests, as well as spontaneous, cocaine-induced and cocaine-conditioned motor activity. *Homer2^AA/AA^* manifested lower behavioral signs of a anxiety-like state within the elevated plus-maze and light-dark box tests that align with the phenotype of *GRM5^R/R^* KI mice with reduced steady state Homer binding (Table 3; Cozzoli et al., 2009; Guo et al., 2016) and *GRM5^AA/AA^* KI mice with deficient signal-regulated mGlu5 interactions with Homer (Table 3; Campbell et al., 2019; Park et al., 2013). However, distinct from either of these *GRM5* KI lines, *Homer2^AA/AA^* mice were resilient to the conditioned aversive properties of high-dose cocaine as determined under both place-conditioning and taste-conditioning procedures. Our results support a model where high doses of cocaine strongly activate striatal neurons that leads to CaMKIIα activation, Homer2 phosphorylation and disruption of mGlu5-Homer complexes. The disruption of mGlu5-Homer in the striatum (and perhaps other brain regions implicated in the affective/motivational properties of cocaine) then changes the valence of high dose cocaine from rewarding to aversive. Because CaMKIIα phosphorylation of Homer2 also reduces its affinity for other interactors such as drebrin and mGlu1, Homer2 complexes with other proteins may also be affected by high dose cocaine and contribute to cocaine aversion.

**Table 3:** Comparison of behavioral phenotypes expressed by *Homer2^AA/AA^*, *GRM5^R/R^*, and *GRM5^AA/AA^* mice

	**WT vs *Homer2^AA/AA^***	**WT vs *GRM5^R/R^***	**WT vs *GRM5^AA/AA^***
mGlu5-coupling	↑ (activity-dependent)	↓ 1 (steady-state)	↓ 3 (activity-dependent)
Acoustic Startle (Fig. 1; sensorimotor processing)	=	= 2	=
Prepulse inhibition (PPI; Fig. 1; sensorimotor gating)	=	↓^2^	=
Morris water maze acquisition (Fig. 2; spatial learning)	↑	=	↑ (females) = (males)
Morris water maze Probe test (Fig. 2; Table 1; spatial memory)	=	=	= (quadrant) ↑ (platform)
Morris water maze reversal training (Fig. 2; spatial reversal learning)	=	n.d.	n.d.
Elevated plus maze open arm time and entries (Fig. 3*A,B*; anxiety; agoraphobia)	↑	↑^2^	↑
Light-Dark box (Fig. 3*C*) latency and light entries (anxiety; photophobia/agoraphobia)	↓ (latency) = (entries)	n.d.	n.d.
Novel object reactivity (Fig. 3*D–F*) latency and contacts (anxiety; neophobia/agoraphobia)	=	↑ (latency) ↓ (contacts)	=
Marble burying test (Fig. 3*G*) latency and # buried (anxiety; neophobia)	=	n.d.	n.d.
Forced swim test (Fig. 3*H,I*) (active vs passive coping)	=	=^2^	Less floating (active coping)
Acute cocaine locomotion (Fig. 4*A*)	=	= 3	↑ (10 mg/kg)^3^
Sensitized cocaine locomotion (Fig. 4*B*)	=	= 3	↓ (10 and 30 mg/kg)^3^
Dose-response function for cocaine-induced place-preference (Fig. 5*A,B*)	Shift to the right	No change	Shift to the left^4^
Cocaine taste aversion (Fig. 5*D,F*)	↓	n.d.	=

↑ indicates an increase versus WT; ↓ indicates a decrease versus WT; = indicates no genotypic difference; n.d. indicates not determined.

^1^Cozzoli et al. (2009).

^2^Guo et al. (2016).

^3^Park et al. (2013).

^4^Campbell et al. (2019).

### Preventing steady-state, or activity-dependent changes in, mGlu5-Homer interactions induces signs of hypoanxiety

mGlu5 activity has long been implicated in the neuropathology of depression and other stress-related disorders (cf. Spooren et al., 2010; Peterlik et al., 2016; Kasten et al., 2019; Dogra and Conn, 2021). Aligning with this, adult *GRM5* KO mice exhibit a hypoanxious phenotype (Olsen et al., 2010; Inta et al., 2013; Xu et al., 2021) and negative allosteric modulators of mGlu5 exhibit anti-depressive and anxiolytic effects (Peterlik et al., 2016; Kasten et al., 2019; Dogra and Conn, 2021). mGlu5 scaffolding by Homer proteins and regulation of this scaffolding may be pivotal for the capacity of this receptor to gate affective states in response to stressors as mutations that either abrogate steady-state mGlu5-Homer interactions (e.g., *GRM5^R/R^* KI or *Fmr1* KO mouse; Guo et al., 2016; Table 3) or prevent activity-dependent or signal-regulated changes in mGlu5-Homer binding avidity (e.g., *Homer2^AA/AA^*, *GRM5^AA/AA^* KI and *Homer1a* KO mouse; Datko et al., 2017; Campbell et al., 2019) all exhibit certain signs of hypoanxiety in behavioral test batteries for negative affect, although the specific behavioral outcome affected varies across the different mutant lines (see summary in Table 3). Although Homer2 expression is upregulated within mesocorticolimbic structures by stressors (Ary et al., 2007; Quadir et al., 2016), the affective phenotype of *Homer2* KO mice does not diverge from WT controls (Szumlinski et al., 2004, 2005a). In contrast, *Homer1* KO mice, lacking both the constitutively expressed and inducible Homer1 isoforms, exhibit a pronounced spontaneous negative affective state (Szumlinski et al., 2004, 2005a; Lominac et al., 2005; Jaubert et al., 2007; Wagner et al., 2014, 2015). Based on the distinct affective phenotypes of *Homer1* versus *Homer2* KO mice, one might infer that the hypoanxious phenotype exhibited by *GRM5^R/R^* KI, *GRM5^AA/AA^* KI, *Homer1a* KO, and *Fmr1* KO mice reflect reduced mGlu5-Homer1a binding or deficits in regulated mGlu5-Homer1 binding.

However, we show herein that preventing the CaMKIIα-dependent phosphorylation of Homer2 and dissociation from its interactors is also sufficient to reduce certain signs of anxiety-like behavior in response to acute stressors (see Table 3). *Homer2^AA/AA^* mice exhibit WT-levels of basal Homer2 expression (Fig. 7*A*). Although the lack of any affective phenotype in *Homer2* KO mice was originally ascribed to compensation by Homer1 scaffolding (Szumlinski et al., 2005a), the present data for *Homer2^AA/AA^* implicate stressor-induced changes in CaMKIIα-mediated phosphorylation of Homer2, rather than changes in steady-state Homer2 expression, as important for gating behavioral reactivity to stressors. Indeed, mice with null deletions of the gene encoding CaMKIIα (*CAMK2A* KO; Chen et al., 1994), as well as KI mice lacking the T286 autophosphorylation site on CaMKIIα required for its activation (Easton et al., 2011; Gustin et al., 2011), exhibit reduced signs of anxiety-like behavior (Chen et al., 1994) and infusion of the CaMKII inhibitor atCN21 peptide reduces elevated signs of a negative affective state in rats with global cerebral ischemia (Ahmed et al., 2017). Taken together, the results of these latter studies, coupled with our present data from *Homer2^AA/AA^* mice, implicate stressor-induced CaMKIIα-mediated phosphorylation of Homer2, and its dissociation from interactors, as a molecular switch driving anxiogenesis. The *Homer2^AA/AA^* mutation also prevents activity-depending interactions with other postsynaptic density proteins, including drebrin and mGlu1 (Guo et al., 2015), and both of these interactors have been implicated in anxiety-like behavior (Spooren et al., 2010; Peterlik et al., 2016; Milanovic et al., 2017). Thus, it will be important in future work to decipher which of these (or other) Homer2 interactions is critical for anxiogenesis of relevance to targeting anxiety disorders.

### Preventing activity-dependent changes in mGlu5-Homer interactions improve MWM performance

CaMKII activity plays a pivotal role in the regulation of synaptic strength underpinning the neural plasticity necessary for normal learning, memory consolidation and recall (cf. Mayford et al., 1996; Giese et al., 1998; Soderling and Derkach, 2000; Miller et al., 2002; Wayman et al., 2008). *Fmr1* KO rats (Tian et al., 2017) and certain strains of *Fmr1* KO mice (Kooy et al., 1996; D’Hooge et al., 1997; Paradee et al., 1999; Dobkin et al., 2000; Van Dam et al., 2000; Baker et al., 2010) exhibit deficits in spatial memory assessed under MWM procedures. The spatial learning deficits exhibited by *Fmr1* KO rodents might reflect reduced scaffolding of mGlu5 by long Homer isoforms within forebrain (Giuffrida et al., 2005), which is rescued by genetic deletion of the short Homer1a isoform (Ronesi et al., 2012) and mimicked by peptide-mediated disruption of mGluR5-long Homer scaffolds in WT mice (Ronesi and Huber, 2008; Ronesi et al., 2012; Tang and Alger, 2015). Herein, the *Homer2^AA/AA^* mutation that prevents CaMKIIα-dependent dissociation of Homer2 from its interactors (Guo et al., 2015; Fig. 7*B*) facilitated the MWM acquisition (Fig. 3*A*). A facilitation of MWM learning was also reported previously in *Homer1a* KO mice (Datko et al., 2017) that exhibit a relatively higher proportion of long Homer binding to interactors following neuronal activity (Tu et al., 1998). Together, these studies suggest that the capacity of long Homer proteins to maintain protein scaffolds (presumably with mGlu5) during the neuronal activity that accompanies spatial learning enables the synaptic plasticity facilitating behavioral outcomes. Implicating an important role for activity-dependent Pin1-mediated isomerization of mGlu5 in this mechanism is the observation that *GRM5^AA/AA^* KI female mice also exhibited facilitated MWM acquisition (Fig. 3*B*,*C*) and both male and female *GRM5^AA/AA^* KI mice entered the former platform location more times during the memory probe test (Table 1). Although it is not clear at the present time why the effect of the *GRM5^AA/AA^* mutation on MWM acquisition was female selective, it is worth noting that *GRM5^R/R^* mice exhibit WT-levels of MWM performance (Fig. 3). Although the *GRM5^R/R^* mutation abrogates mGlu5 binding of all Homer isoforms (Cozzoli et al., 2009; Guo et al., 2016), these mutants exhibit constitutive mGlu5-driven protein synthesis rates, translation initiation, and both ERK and mTORC1 signaling (Guo et al., 2016). *GRM5^R/R^* mice also exhibit intact signal-induced, Pin1-directed, isomerization of mGlu5 (Park et al., 2013), all of which might compensate for reduced mGlu5-Homer binding to maintain cognitive function. It should be noted that while *Homer2^AA/AA^* and *GRM5^AA/AA^* exhibit a common MWM phenotype to implicate mGlu5 as a key Homer2-interacting protein gating spatial learning, the *Homer2^AA/AA^* mutation affects activity-dependent binding to other interactors (Guo et al., 2015). In this regard, Homer2 interactions with drebrin are particularly intriguing as this F-actin binding protein is central to memory-related hippocampal synaptic plasticity (Jung et al., 2015), is downregulated in postmortem brains of individuals with Alzheimer’s disease (Rao et al., 2011; Counts et al., 2012), and mimicking drebrin down-regulation within hippocampus accelerates cognitive impairment in a genetic mouse model of Alzheimer’s disease (Y. Liu et al., 2017).

### Preventing activity-dependent changes in mGlu5-Homer interactions spare cocaine reward but eliminate cocaine aversion

The capacity of Homer proteins to regulate behavioral sensitivity to cocaine was recognized two decades ago (Ghasemzadeh et al., 2003) and studies of null *Homer1*, *Homer2* and *Homer2* mutant mice provided corroborative evidence that the loss of Homer1 or Homer2 expression produces a cocaine “presensitized” phenotype, characterized by increased locomotor responsiveness to acute cocaine, heightened cocaine-conditioned reward, higher cocaine reinforcement, “cocaine-like” glutamate anomalies within the nucleus accumbens, including reduced synaptic membrane expression of group 1 mGlu and NMDA receptors (Szumlinski et al., 2004, 2005b; Lominac et al., 2005). Withdrawal from repeated cocaine exposure also increases the relative expression of long Homer2 versus Homer1 isoforms within prefrontal cortex (Ary and Szumlinski, 2007; Gould et al., 2015; Chiu et al., 2021), which not only elevates prefrontal cortex extracellular glutamate (Ary et al., 2013) to drive cocaine relapse-like behavior (Gould et al., 2015; Shin et al., 2018), but is sufficient to produce, in cocaine-naive animals, many “cocaine-like” biochemical changes within the nucleus accumbens known to increase behavioral sensitivity to the drug, including a reduction in Homer1 and Homer2 expression (Ary et al., 2013).

The first direct evidence supporting mGlu5-Homer interactions as important for cocaine-induced behavioral plasticity was derived from studies of *GRM5^AA/AA^* mice, which (akin to *Homer1* and *Homer2* KO mice; Szumlinski et al., 2004) also exhibit increased acute cocaine-induced locomotor activity, but fail to exhibit cocaine-induced behavioral or neurochemical sensitization on repeated cocaine treatment, implicating signal-regulated mGlu5-Homer binding as important for cocaine-induced neuroplasticity (Park et al., 2013). Consistent with this, rats exhibiting a time-dependent intensification or incubation of cue-elicited cocaine-craving exhibit reduced Homer1 and Homer2 scaffolding of mGlu5, but not mGlu1, within the nucleus accumbens; however, attempts to maintain Homer scaffolding by virus-mediated overexpression of either long Homer isoform failed to affect cocaine self-administration or self-craving (Loweth et al., 2014). Consistent with this, mimicking the cocaine-induced dissociation of mGlu5-Homer interactions with the *GRM5^R/R^* mutation does not alter either the acute or sensitized locomotor response to cocaine (Park et al., 2013), nor does it alter the expression of a cocaine-conditioned place-preference (Fig. 6*B*). While such findings argue perhaps a more important role for Homer binding to other interactors in gating the rewarding and reinforcing properties of cocaine, *GRM5^AA/AA^* mice, with impaired Pin1-directed isomerization of mGlu5, and impaired signal-induced regulation of mGlu5-NMDA interactions (Orlando et al., 2009; Park et al., 2013), exhibit a robust cocaine-conditioned place-aversion to cocaine doses that elicit a place-preference in WT mice (Campbell et al., 2019). As Pin1-directed isomerization of mGlu5 is intact in *GRM5^R/R^* mice (Park et al., 2013), the discrepancies in findings between *GRM5^R/R^* and *GRM5^AA/AA^* mice argued that signal-regulated, Pin1-directed, mGlu5 isomerization and consequent changes in mGlu5-Homer binding avidity and/or NMDA receptor regulation, normally enable cocaine-induced neuroplasticity driving the positive motivational/affective properties of cocaine (Campbell et al., 2019). Opposite *GRM5^AA/AA^* mice (Campbell et al., 2019), *Homer2^AA/AA^* mice exhibit cocaine-conditioned place-preference (Fig. 6*A*) and no cocaine-conditioned taste-aversion (Fig. 6*C*) at cocaine doses that elicit, respectively, place-ambivalence and taste-aversion in WT mice. As cocaine taste-conditioning involves a single cocaine injection, these findings demonstrate that preventing Homer2 phosphorylation blocks cocaine aversion in both the acute and drug-experienced state. Importantly, the insensitivity of *Homer2^AA/AA^* mice to cocaine’s aversive properties does not reflect hypersensitivity to the drug’s psychomotor activating properties as *Homer2^AA/AA^* mice exhibited WT-levels of cocaine-induced locomotor activity and stereotypy (Fig. 5). The insensitivity also does not reflect an inability to detect the interoceptive effects of cocaine as *Homer2^AA/AA^* mice exhibited WT-comparable cocaine-induced place-preference at the lower cocaine dose tested. While we did not conduct a dose–response study, high-dose cocaine (20 mg/kg) induced CaMKIIα-directed phosphorylation of Homer2 to dissociate mGlu5-Homer2, and this cocaine-induced dissociation of mGlu5-Homer2 binding was blocked by in *Homer2^AA/AA^* KI mice (Fig. 7). These findings indicate that *Homer2^AA/AA^* mice effectively maintain the capacity of Homer2 to bind interactors in the presence of cocaine-induced neuronal activity. While it will be important in future work to determine whether mGluR-Homer2 interactions are maintained in response to aversive cocaine doses (e.g., 30 mg/kg; Fig. 6), we propose that the polar opposite effects of the *GRM5^AA/AA^* versus *Homer2^AA/AA^* mutations on the expression of cocaine aversion (potentiation vs block, respectively) may simply reflect the opposing consequences of cocaine-induced neuronal activity on mGlu5-Homer2 binding avidity (reduction vs potentiation, respectively). Alternatively, the resiliency of *Homer2^AA/AA^* mice to cocaine aversion may be completely unrelated to mGlu5 binding and reflect a heightening of interactions with other proteins. As insensitivity to the aversive properties of drugs of abuse is a major risk factor for developing a substance use disorder (de Wit et al., 1986; Chait, 1996; de Wit and Phillips, 2012; Riley et al., 2022), delineating the precise interactions impacted by the *Homer2^AA/AA^* mutation may provide critical insight into substance use disorder vulnerability.

In conclusion, using a transgenic mouse model with deficits cocaine-induced uncoupling of mGlu5-Homer2, we demonstrate an important role for Homer2 scaffolding of mGlu5 in regulating cocaine’s aversive properties, without influencing cocaine reward. Findings suggest that environmental factors, to include cocaine exposure, that affect mGlu5-Homer2 scaffolding dynamics may contribute to an individual’s subjective response to cocaine to influence addiction vulnerability.

## Synthesis

Reviewing Editor: Christie Fowler, University of California, Irvine Decisions are customarily a result of the Reviewing Editor and the peer reviewers coming together and discussing their recommendations until a consensus is reached. When revisions are invited, a fact-based synthesis statement explaining their decision and outlining what is needed to prepare a revision will be listed below. The following reviewer(s) agreed to reveal their identity: NONE.

In this study the authors performed behavioral characterizations of several mouse lines carrying point mutations in either in genes encoding mGlu5 or Homer2 proteins, designed to either prevent protein-protein interactions or phosphorylation at key residues. Major findings of the studies show that prevention of Homer2 phosphorylation eliminates place aversion induced by high doses of cocaine. The manuscript represents a large body of work and provides some insight into the role of glutamate signaling and receptor scaffolding in behavioral responses to cocaine. However, some significant concerns need to be addressed.

Major:

1. One of the main conclusions draws from the cocaine CPP differences between groups. However, it appears that the WT littermates of the Homer2AA/AA mice do not show CPP at the higher dose of cocaine (Figure 5 A, 30 mg/kg dose). This needs to be addressed.

2. It would be of benefit to include individual data points on all of the bar graphs to provide a better understanding of the variability in the data sets.

3. The novel object test employed is not standard. Normally animals are placed in an environment with two identical novel objects during a familiarization phase, followed by re-exposure to the same object and replacement of the other with a newer object. The current paradigm only measured responsivity to one object during one test period. A detailed rationale for use of this unusual paradigm needs to be provided. In addition, more detailed descriptions of the actual objects used and verification that the objects had no inherent attractive or aversive qualities is needed, as this would influence object reactivity during the only exposure period.

4. Neocortical slide preparations were obtained from animals at the very early stage of adolescence (P28-P30). It is unclear why this was not performed in adult mice to coincide with the age used in a multitude of behavioral tests assayed.

5. For assessment of stereotypical behavior, mice were assessed for 30 sec only every 10 min during a 60 min test session. Thus, total assessment time was only 180 sec (3 min) out of a 1 hour period, and numerous instances of stereotypical or normal behavior might have been missed. With such a small sampling period, it is therefore unclear what value these data add to the overall study.

6. There are several issues with the graphs that need to be corrected:

a. In Figure 1, all panels, please provide units for startle amplitude on the y-axes

b. In Figure 3, none of the panels have y-axis labels or units

c. In Figs. 4B, 5D, 5F - the term “delta” is used presumably to describe changes in solution in intake or distance traveled, but this is not abundantly clear in the methods section.

7. Different doses of cocaine are used without rationale. It is noteworthy that the Figure 6 data used 20 mg/kg of cocaine, a dose that was not used in the behavioral studies demonstrating significant differences between genotypes. Thus, it is difficult to reconcile the relevance of these data to the behavioral studies presented.

Minor:

1. Based on the manuscript title, it appears that the main findings the authors wish to convey is the loss of cocaine aversion in Homer2AA/AA mice. However, a number of other phenotypes were observed in these and other mouse lines, including altered anxiety-like behaviors in the EPM and marble burying tests in Homer2 and Grm5AA/AA mice as shown in Fig. 3. Thus, the current title may be somewhat self-limiting in terms of summarizing the current data set.

2. In the swim test, what was the temperature of the water used? Also, for the 11-cm diameter cylinder please state that it actually contained water.

3. The term ‘emotional’ should not be used to describe behavioral testing in mice. It is arguably not possible to understand the animal’s emotions. Thus, this term should be removed from the manuscript. Similarly, the description of ‘emotional’ states needs to be modified accordingly. For instance, ‘hypo-anxiety’ should be described as a ‘decreased anxiety-associated state’.
